# Comparative Analysis of Cotton Small RNAs and Their Target Genes in Response to Salt Stress

**DOI:** 10.3390/genes8120369

**Published:** 2017-12-05

**Authors:** Zujun Yin, Xiulan Han, Yan Li, Junjuan Wang, Delong Wang, Shuai Wang, Xiaoqiong Fu, Wuwei Ye

**Affiliations:** 1 State Key Laboratory of Cotton Biology, Institute of Cotton Research of Chinese Academy of Agricultural Sciences, Anyang 455000, China; zujuny@163.com (Z.Y.); lycaas@163.com (Y.L.); wangjj@cricaas.com.cn (J.W.); wangdl@cricaas.com.cn (D.W.); wangshuai_19871201@163.com (S.W.); 2 State Key Laboratory of Crop Biology, College of Agronomy, Shandong Agricultural University, Tai’an 271018, China; genetics@sdau.edu.cn

**Keywords:** microRNAs, miR390, retrotransposons, TAS3, ARF4, tasiRNA-ARFs

## Abstract

Small RNAs play an important role in regulating plant responses to abiotic stress. Depending on the method of salt application, whether sudden or gradual, plants may experience either salt shock or salt stress, respectively. In this study, small RNA expression in response to salt shock and long-term salt stress in parallel experiments was described. Cotton small RNA libraries were constructed and sequenced under normal conditions, as well as sudden and gradual salt application. A total of 225 cotton microRNAs (miRNAs) were identified and of these 24 were novel miRNAs. There were 88 and 75 miRNAs with differential expression under the salt shock and long-term salt stress, respectively. Thirty one transcripts were found to be targets of 20 miRNA families. Eight targets showed a negative correlation in expression with their corresponding miRNAs. We also identified two TAS3s with two near-identical 21-nt trans-acting small interfering RNA (tasiRNA)-Auxin Response Factors (ARFs) that coaligned with the phases D7(+) and D8(+) in three *Gossypium* species. The miR390/tasiRNA-ARFs/ARF4 pathway was identified and showed altered expression under salt stress. The identification of these small RNAs as well as elucidating their functional significance broadens our understanding of post-transcriptional gene regulation in response to salt stress.

## 1. Introduction

Salt stress is the exposure of plants to high salinity, the main component of which is Na^+^ ions. It affects various aspects of plant growth and development, such as inhibition of enzymatic activities and reductions in photosynthetic rates [1]. Depending on the method of NaCl application, whether in a single step or gradual, plants experience either salt shock or salt stress mainly with ionic stress component, respectively [2]. Salt shock and salt stress mainly with ionic stress component are two distinct phenomena. Salt shock immediately induces osmotic shock when roots come into contact with solutions containing unfavourably high concentrations of salts in hydroponic systems or in soil [3]. However, the concentration of Na^+^ reaches a certain toxic level in cell protoplasts of shoots under long-term salt stress [4]. Osmotic stress results in strong, rapid changes in the expression of genes with antioxidant system function, and a few changes in ion-responsive genes [5]. Many genes with altered expression under long-term salt stress maybe involved in responses to toxic cellular concentrations of Na^+^ ions. There are very few studies in which the effects of salt shock and long-term salt stress are described in parallel experiments [6,7]. A more detailed analysis of the process should broaden our understanding of the molecular events of the mechanisms involved in salt stress response. 

The wave of gene activation is a consequence of stepwise transcriptional, post-transcriptional and post-translational regulation [8]. MicroRNAs (miRNAs), an abundant class of short non-coding (21–23 nt) RNAs, are generated from single-strand hairpin RNA precursors [9,10]. They regulate gene expression by binding to targeted mRNA sequences in a perfect or near-perfect complementary site. After that, they lead to targeted sequence cleavage or, in at least a few cases, translational repression in plants [11]. Many processes, such as leaf development, auxin signaling, phase transition, flowering and genome maintenance are regulated in similar ways by different miRNAs [12]. Another type of small RNAs in plants is small interfering RNAs (siRNAs), a class of double-strand RNAmolecules that also act in RNA interference (RNAi)-related pathways [11]. This encompasses heterochromatin-associated siRNAs and trans-acting siRNAs (tasiRNAs), repeat-associated siRNAs (rasiRNAs) and natural antisense transcript siRNAs (nat-siRNAs) [13]. 

Recently findings have firmly established a role for miRNAs in plant stress responses [14,15]. For example, Arabidopsis miR393 is strongly up-regulated by cold, dehydration, NaCl and abscisic acid (ABA) treatments [16]. It targets the F-box auxin receptors Transport Inhibitor Response Protein 1 (TIR1) and Auxin Signaling F-box Protein 1 (AFB1) [17]. Over-expression of *osa*-*MIR393* leads to more tillers, early flowering and less tolerance to salt and drought [18]. Xie et al. [19] investigated the transcriptional profiles of cotton miRNAs during exposure to drought and salinity stress. miR413 and miR440 were found to be expressed specifically in salt treatments. Some of miRNAs were also found to be expressed differentially in terms of dose dependence and tissue dependence under stress conditions. In cotton roots exposed to salinity, miR399 shows down-regulated expression levels with the increase of salt concentration [20]. The expression levels of miR156, miR157 and miR172 were up-regulated at 0.25% NaCl and then down-regulated at higher salinity concentrations [20]. Many discoveries that stress can regulate miRNA levels, coupled with the identification of stress-associated genes as miRNA targets provided clues about the role of miRNAs in stress responses [21]. For example, *PHO2* (phosphate responsive mutant 2), encoding E2 conjugase, has been verified as a target of miR399 in model plant species [22]. In addition, constitutive miRNAs in maize or cotton were shown to be capable of genotype-specific expression in response to salt stress [23,24]. Therefore, a more detailed analysis of the kinetics of miRNAs and their target genes could be helpful in obtaining better insights into the mechanisms and roles of miRNAs in salt-regulatory networks. Cotton (*Gossypium hirsutum* L.) is considered to be relatively tolerant to salinity [25]. Its growth and productivity are also adversely affected by salt stress. In the past decades, much progress has been made in unraveling the complex stress response mechanisms, and considerable information has become available on the identification of stress responsive genes. However, little is known about the roles of small RNAs in response to different salt stress phases. In this study, the small RNAome, degradome and transcriptome were studied to investigate miRNA expression profiles in response to the effect of sudden and gradual salt application. These data provide new and valuable insights into the expression profiles of miRNAs, other small RNAs and target genes, and their relationships under salt stress. 

## 2. Materials and Methods

### 2.1. Plant Materials and Growth Conditions

The salt-tolerance cotton cultivar ‘SN-011’ was used in this study. Seeds were sterilized and germinated in vermiculite. Growth conditions were 30/22 °C day/night temperature, 55–70% relative humidity and a 14/10 h light/dark cycle under 450 μmol m^−2^·s^−1^ light intensity. At the two-leaf stage, healthy seedlings were placed in pots containing aerated Hoagland nutrient solution. Plants were cultured under non-saline conditions for 10 d to ensure full establishment before starting the salinity treatments. The pH was maintained close to 6.9 by adding H_2_SO_4_ or KOH as required. Seedlings were randomly divided into two groups that were treated with salt stress. The remaining seedlings served as control. In all experiments 15 plants were used for each treatment. For the sudden salt application, seedlings were suddenly transferred from normal growth solution into a 150 mM NaCl solution. Leaves were harvested after exposing the seedlings to salt stress for 2, 4 and 8 h. For the gradual salt application, seedlings were exposed to salinity by adding NaCl to the growth medium in 50-mM increments every 12 h, until a final concentration of 150 mM was reached. After exposing the seedlings to salt stress for 1, 3 and 5 d, leaves were harvested directly into liquid nitrogen and stored at −80 °C for subsequent use.

### 2.2. Measurement of Na^+^ Contents and Proline Level

Leaves were dried for 72 h at 70 °C, and the dry weight measured. Dried leaves were then digested with 0.1 M HNO_3_, and Na^+^ contents were determined by inductively coupled plasma-optical emission spectrometry (ICP-OES) (Optima 2100 DV; Perkin-Elmer, Wellesley, MA, USA) according to the manufacturer’s instructions. Proline level was measured using commercial assay kits (Jiangsu Suzhou Keming Bioengineering Institute, Suzhou, Jiangsu, China) according to the manufacturer’s instructions. Three independent biological replicates were performed.

### 2.3. Total RNA Isolation andConstruction of Small RNA and Degradome Libraries

Total RNA was isolated using a modified CTAB method with isopropanol instead of lithium chloride for RNA precipitation and purified using RNeasy columns (Qiagen, Hilden, Germany) [26]. RNA quantity and quality were checked by Agilent 2100 Bioanalyzer. The small RNA digitalization analysis based on HiSeq high-throughput sequencing takes the sequencing by synthesis (SBS), which is useful due to its small requirement of sample quantity, high throughput, high accuracy with a simply operated automatic platform. Small RNA libraries were constructed as previously described [27,28]. In brief, total RNA was separated by 15% denaturing polyacrylamide gel electrophoresis to recover the population of small RNAs. The extracted RNAs were then dephosphorylated using alkaline phosphatase and ligated to 5′- and 3′-RNA adaptors by T4 RNA ligase. The adaptor-ligated small RNAs were subsequently transcribed to single-strand cDNA using SuperScript II Reverse Transcriptase. After PCR amplification, PCR products were purified and subjected to SBS sequencing with an Illumina Solexa genome analyzer II at the BGI (Beijing Genomics Institute, Shenzhen, China).

Degradome sequencing provides a comprehensive means of analyzing patterns of RNA degradation. The libraries were constructed as previously described [29]. In brief, poly(A^+^) RNA molecules were isolated from 150 μg of total RNA using the Oligotex mRNA kit. Subsequently, the 5′-phosphate of the poly(A^+^) RNA was ligated to a 5′-RNA oligonucleotide adaptor containing a *Mme*I recognition site by T4 RNA ligase. The products of a reverse transcription reaction were amplified by five PCR cycles and were then digested with *Mme*I and ligated to a 3′ double-DNA adaptor. The ligation products were purified and amplified by 20 PCR cycles and gel-purified for SBS sequencing.

### 2.4. Transcriptome Sequencing andde novoAssembly

To maximize the transcript representation in a broad range of biological processes, the total RNAs harvested at 0 h, 4 h and 5 d after salt treatment were pooled in an equal fraction ratio. Poly(A^+^) mRNAs were isolated using beads with Oligo(dT). Fragmentation buffer was then added to interrupt mRNA into short fragments. The first-strand cDNA was synthesized using random hexamer-primer. The second-strand cDNA was synthesized using buffer, dNTPs, RNaseH and DNA polymerase I, respectively. Short fragments were purified and resolved with Elution buffer for end reparation and tailing A. Sequencing adapters were linked with short fragments. After that, suitable fragments were selected by agarose gel electrophoresis, and were used for the PCR amplification as templates. The library was sequenced using Illumina HiSeq™ 2000 (Illumina, CA, USA). Dirty raw reads which contained adapters, unknown or low quality bases were discarded. Transcriptome de novo assembly was carried out with Trinity, a short reads assembling program [30]. Briefly, Trinity firstly combined reads with certain length of overlap to form contigs. Then the contigs with paired-end reads were connected to form unigene sequences that could not be extended on either end. For function annotation, unigene sequences are aligned by blastx to protein databases of *G. hirsutum* (*e*-value < 0.00001), and aligned by blastn to CDS databases of *G. hirsutum* (*e*-value < 0.00001), retrieving proteins with the highest sequence similarity with the given Unigenes along with their protein functional annotations.

### 2.5. Analysis of Small RNA and Degradome Sequencing Data

The very basic figure from small RNA and degradome sequencing is converted into sequence data by the base-calling step. As for the small RNA library, raw sequencing reads were processed into clean full-length reads by the BGI small RNA pipeline [28]. After the raw sequence reads were obtained, adapter–adapter sequences, low quality tags, contaminants and insert sequences beyond 15–30 nt were filtered. The remaining sequences were aligned to Rfam and GenBank databases to annotate rRNA, tRNA, snRNA, snoRNA and those containing polyA tails [31]. Next, sequence tags were searched against the miRNA database, miRBase (Release 20.0, June 2013), in order to identify similar miRNAs. All plant miRNAs were downloaded and were utilized as dataset to perform alignment searches. The reads that were shorter than 24 nt, and had more than one copy in a read library, either shorter/longer or contained up to two mismatches, and no match to known non-coding RNAs, were tested whether they are miRNAs [32]. The small RNA sequences which mapped on unigenes from transcriptome sequencing were used to predicted novel potential miRNAs. The novel miRNAs were identified using the MIREAP program developed by the BGI [33]. For this procedure, the occurrence of both a miRNA and a miRNA* in the deep sequencing data was the basis to predict novel miRNAs. The approximately 23 nt potential miRNA sequence is not located on the terminal loop of the hairpin structure. ThemiRNA has less than six mismatches with the opposite miRNA sequence (miRNA*) on the other arm [34]. The filtered pre-miRNA sequences were folded again using MFOLD software and selected manually [35,36]. MiRNAs expression abundance was analyzed by counting the number of transcripts per million (TPM) clean reads in libraries. We first normalized the read density measurement and then used *p*-value < 0.01 and the absolute value of |log_2_^Ratio^|>1 as a threshold to judge the statistical significance of miRNA expression. From the three data sets, plenty of miRNAs were found to be differentially expressed between libraries.

Degradome sequencing takes the SE50 sequencing strategy and produces 49-nt raw reads. The 3′-adaptor was trimmed before bioinformatics analysis to get real degradome fragments with length of 20–21 nt. Data filtering was carried out to obtain high quality reads as clean reads. The unique tags and total tags were first analyzed. Then, the degradome tags were annotated with rRNA, scRNA, snoRNA, snRNA and tRNA from GenBank and Rfam, and selected out from unannotated tags. The degradome sequences with a single base representing over 70% of the bases were regarded as polyN and tossed. To map every unique degradome tag to only one of these annotations, we followed the priority rule: Rfam > GenBank > PolyN. Unannotated tags were mapped to reference genes (cDNA). The tags mapped to cDNA_sense were used to predict cleavage sites. After a series of annotation of the degradome sequence, the degradation mRNA obtained go through comparison with miRNA to find mRNA-miRNA pairing. Sequences with less than 4 nt mismatches compared with the query miRNA sequences were selected. The G:U pair was counted as 0.5 mismatch. Cleavages induced by different miRNA sequences were regarded as different cleavage events. The reference genes which were predicted to degradome tags were annotated. The T-Plot figure was constructed to show the tag distributions of one gene. 

### 2.6. qRT-PCR Analysis

For cDNA synthesis,1 μg of total RNA was used. First-strand cDNA was synthesized using a PrimeScript RT reagent kit with gDNA eraser (Takara, Dalian, China). Premix Ex TaqTM II(TaKaRa)was used for real-time quantitative PCR (qRT-PCR). Gene-specific primers are shown in Table S1. qRT-PCR was undertaken with a 7500 Fast Real-Time PCR system (Applied Biosystems Inc., Carlsbad, CA, USA). The 25-μL reaction solutions contained 12.5 μL 2XSYBR Premix Ex TaqTM II, 1 μL forward primer, 1 μL reverse primer, 2 μL DNA template and 8.5 μL H_2_O. For the mature miRNAs, RT-PCR employed a stem-loop primer to detect miRNAs. The primers were based on those described by Chen et al. and Varkonyi–Gasic et al. [37,38]. Their 3′-ends, which were complementary to the six nucleotides at the miRNA 3′-end, were combined with the 44-nt sequence to form a stem-loop structure.The stem-loop reverse transcription reactions were performed using M-MLV Reverse Transcriptase (Invitrogen) according to the supplier’s manual. U6 was used as a reference for small RNA expression validation [39]. The expression levels of target transcripts were normalized using the housekeeping gene *UBQ7* as the reference control. Three biological replicates were used for qRT-PCR validation. The 2^−^^ΔΔT^ method was used to calculate relative expression levels of salt-responsive miRNAs and target genes.

### 2.7. Statistical Analysis

The statistical significance of Na^+^ contents, proline level, expression profiles of miRNAs and genes was compared by one-way analysis of variance (ANOVA), followed by a Duncan’s multiple range test. All data were analyzed by SPSS10.0 (SPSS Inc., Chicago, IL, USA) software. *p* values of less than 0.05 were considered to be statistically significant.

## 3. Results

### 3.1. Deep-Sequencing of sRNAs under Salt Stress

**Figure 1:**
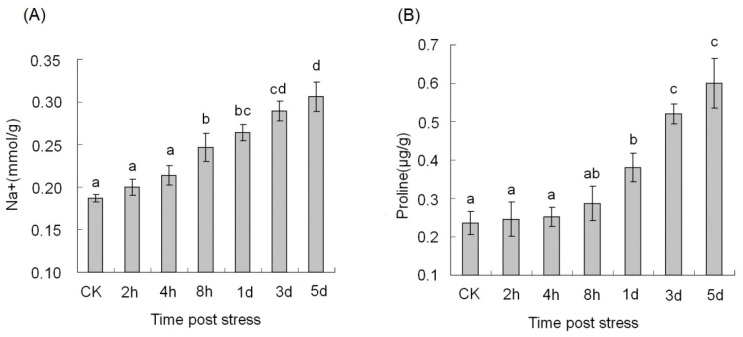
Measurement of Na^+^ content and proline level under salt stress. (**A**) Na^+^ content; (**B**) Proline content. The different letters present on the columns indicate significant differences at *p* < 0.01 among the control samples (CK), 4-h and 5-d salt-treatment samples. Error bars represent standard error of the mean.

Cotton seedlings were randomly divided into two groups to experience two types of salt treatment. One group was suddenly transferred from normal growth solution into 150 mM NaCl solution, experienced salt shock for 2 h, 4 h and 8 h. The remaining seedlings were employed gradual applications of salt, by adding NaCl to the growth medium in 50-mM increments every 12 h, until a final concentration of 150 mM was reached. They were subjected to long-term salt stress for 1 d, 3 d and 5 d. We measured Na^+^ and proline content of seedling leaves under salt stress treatment. The Na^+^ contents in cotton leaves showed an overall increasing trend with the increase of processing time (Figure 1). The 5-d samples showed 1.70-fold higher Na^+^ content than the control samples (CK). No significant difference in Na^+^ content was detected between 8-h samples with sudden NaCl application and 1-d samples with gradual NaCl application. In the 2-h, 4-h and 8-h samples with sudden NaCl application, the proline level was close to those observed in control samples. The content of proline increased after 1 d salt stress treatment under gradual salt application conditions. Based on these results, we selected two time-points (4 h and 5 d) to indicate the effect of sudden and gradual salt application, respectively, for further analysis of salt stress in cotton.

Three separate cDNA libraries of small RNAs were constructed using total RNA obtained from CK and the 4-h and 5-d salt-treatment samples. The small RNA digitalization analysis takes the SBS-sequencing by synthesis. Sequencing raw data were available at Gene Expression Omnibus (GEO: GSE69702). A total of 57,370,554 sequence reads were obtained from the three libraries. After removing the 3p- and 5p-adapter sequences and filtering out low quality ‘n’ sequences, there were 17,026,070, 15,731,477 and 22,590,391 clean reads remaining in the CK, 4-h and 5-d samples, respectively (Table 1). Further analysis identified a total of 12,837,490 unique sequence tags in the three libraries. Approximately 78% (10,023,245) of the unique tags corresponded to singletons. In all three libraries, the majority of the small RNA sequences were 21 nt, followed by 24 nt. The 4-h sample showed higher 21-nt small RNA abundance (48.86%) than CK (40.63%). The frequencies of 21- and 24-nt small RNAs were similar for CK and 5-d samples.

### 3.2. Small RNA Annotation and miRNA Identification

In order toinvestigate the repertoire of miRNAs in cotton, the reads in the small RNA libraries were first mapped to protein-coding and structural RNA-coding RNAs (rRNAs, tRNAs, snRNAs and snoRNAs) in the Rfam and GenBank databases (Figure S1). Thereafter, the remaining sequences were aligned with all known miRNAs in the central miRNA Registry Database, miRBase database. As a result, a total of 201 conserved miRNAs were identified. They were classified into 88 families on the basis of sequence similarity. Among these conserved miRNA families, miR156 and miR166 contained 11 and 12 members, respectively. miR160, miR171, miR172 and miR396 contained nine members; miR167 and miR399 contained seven members; miR159, miR164 and miR390 contained five members; and miR168, miR169, miR319, miR393 and miR482 contained four members. These miRNA families were evolutionarily conserved and had homologs in more than 14 other plant species. However, 59 miRNA families, such as miR391, miR403 and miR822, comprised only one member and showed a low level of conservation.

miRNA genes mostly originate from independent transcriptional units. Transcriptome sequencing was performed to identify primary transcripts of novel miRNAs. The transcriptome raw data have been deposited in the National Center for Biotechnology Information (NCBI) Sequence Read Archive under SRX2865307. We obtained 52,170,788 reads and assembled them into 159,520 contigs. A total of 81,114 unigenes were identified (Table 1), among which 55,689 were annotated in the NCBI non-redundant protein database and 34,147 in the Swiss-Prot database. The remaining non-encoding sequences were used to predict novel miRNAs based on their secondary structure. As a result, 85 candidate novel miRNAs were detected. For 24 of them, the complementary miRNA* species were cloned in libraries, providing an indication of precise excision from the stem-loop precursor. The lengths of these newly identified miRNAs varied within 21–23 nt (Table 2). The predicted hairpin structures of the pre-miRNAs were 76–262 nt, with the majority (69.23%) 90–150 nt (Figure S2). Each miRNA-precursor had high minimal folding free energy index (MFEI) values (0.64–1.74 with an average of about 1.02). The values were significantly higher than that for tRNAs (0.64), rRNAs (0.59) and mRNAs (0.62–0.66) [40].

### 3.3. miRNA Expression Patterns under Different Salt Stress Treatment

miRNAs in small RNA libraries had a very broad range of expression. A few conserved miRNA families, such as miR156, miR157, miR166, miR167 and miR168, showed extraordinarily high expression levels in three libraries. miR157 was the most abundant, with 4,217,486, 5,086,032 and 4,576,202 reads in CK, 4-h and 5-d libraries, respectively. A few less-conserved miRNA families, such as miR829, miR858, miR2118 and miR7490, showed relatively lower expression levels, represented by <10 reads. The results were in agreement with previous studies reporting that less-conserved miRNAs were represented at relatively lower levels than conserved miRNAs. Moreover, different members in the same miRNA family also had significantly different expression levels. For instance, the abundance of miR156 members varied within 7–580,406 reads. The varied frequency of miRNAs suggested their distinct regulatory roles in response to salt stress. 

**Figure 2:**
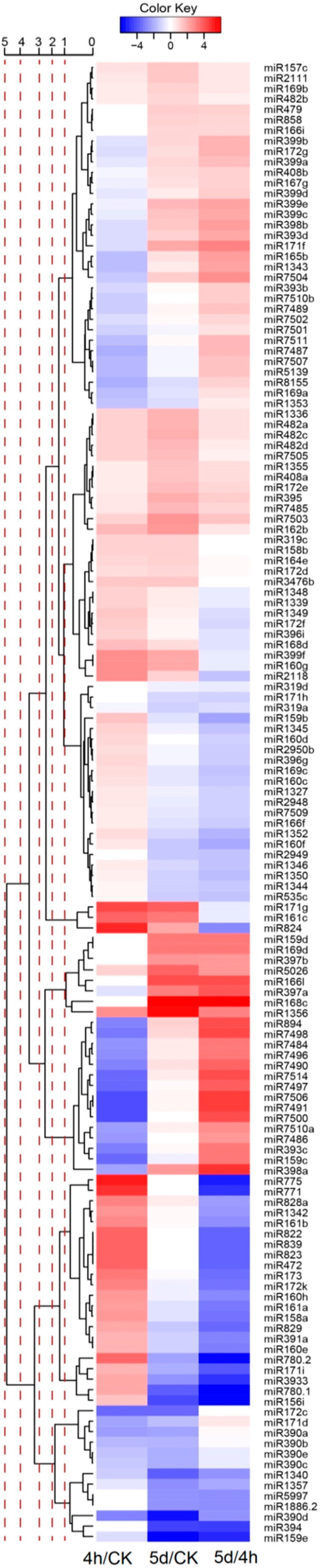
Differentially expressed miRNAs between libraries.

**Figure 3:**
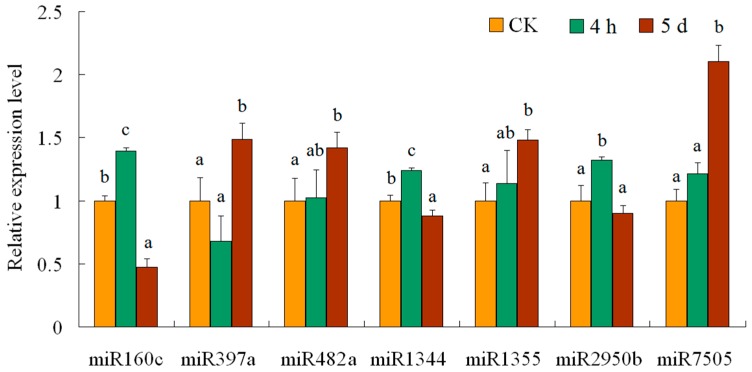
Real-time quantitative PCR validated expressions of seven miRNAs. The amount of expression was normalized to the level of U6. The normalized miRNA levels in control were arbitrarily set to 1. The different letters of each miRNAs present on the columns indicate significant differences at *p* < 0.05 among the CK, 4-h and 5-d samples. Error bars represent standard error of the mean.

miRNA expression abundance in datasets was analyzed by counting the number of transcripts per million (TPM) clean reads in libraries. We first made a comparative analysis of miRNA expression between CK and 4-h libraries (Figure 2). A total of 78 conserved and ten novel miRNAs were differentially expressed between the two samples. Among them, 37 miRNAs were down-regulated and 51 up-regulated. miR780 and miR7498 had the highest down- and up-regulated fold changes, respectively. Next, we compared the normalized expression levels of miRNAs from CK and 5-d libraries, and found 75 miRNAs differentially expressed between the two samples. Among them, 45 conserved and three novel miRNAs, including miR1336, miR1355 and miR1356, were up-regulated in the 5-d compared with CK sample. There were 23 conserved and four novel miRNAs, including miR1340, miR1344, miR1350 and miR1357, down-regulated in the 5-d sample. The most abundant miRNA with differential expression was miR1344, which exhibited a high level (read count 149,649) in CK sample but was less expressed in 5-d library (read count 92,410). Under the two types of salt treatment, a total of 141 miRNAs showed differential expression between all salt treatment and CK samples. Among them, 20 and nine miRNAs showed consistent up- or down-regulation, respectively. Six miRNAs, including miR472, miR771, miR775, miR822, miR823 and miR839, were specifically expressed in the 4-h sample. Three miRNAs, including miR159d, miR166l and miR169d, were specifically expressed in the 5-d sample. These miRNAs had less accumulation of about 2–10 counts. To confirm the expression patterns of the differentially expressed miRNAs, ten miRNAs were selected for a stem-loop RT-PCR experiment (Figure 3). Stem-loop RT-PCR has become the method of choice due to its sensitivity and robust quantification of miRNA levels [41]. Dissociation curve analysis revealed single peaks for the selected primer sets (Figure S3). Real-time quantitative PCR results indicated that seven miRNA showed similar expression patterns to the sequencing results. There was no significant difference between the expression levels of miR482a and miR1355 in the CK and 4-h samples. The remaining three miRNAs tested showed no detectable expression, an observation possibly related to their low accumulation or lower primer set efficiency.

### 3.4. Retrotransposons and Their Endogenous siRNAs

**Figure 4:**
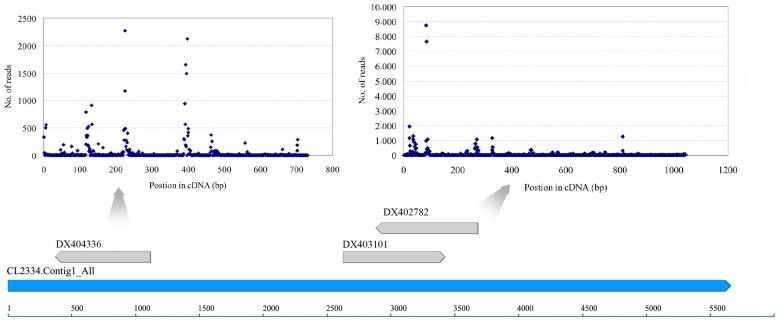
Distribution of small RNAs on three expressed retrotransposons.

**Figure 5:**
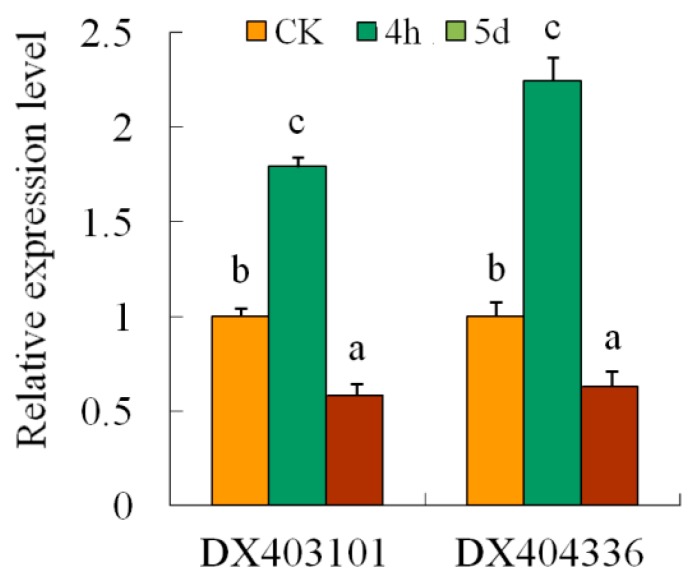
Relative expression levels of two retrotransposons under salt stress. The different letters of each retrotransposons present on the columns indicate significant differences at *p* < 0.05 among the CK, 4-h and 5-d samples. Error bars represent standard error of the mean.

There are 5578 *Gossypium* retrotransposon elements deposited in NCBI. In this study, 36 retrotransposons were found in transcriptome sequencing data. Ten retrotransposon sequences were identical to or had some mismatches with transcripts. For example, three genomic survey sequences, *DX403101*, *DX402782* and *DX404336* were mapped on unigene *CL2334.Contig1* (Figure 4). They were defined as Gorge1 gypsy-like, copia-like and unknown type retrotransposons by Hawkins [42]. Our research showed that some small RNAs had a perfect match with retrotransposon sequences. siRNA is a 21–24 nt long double-strand RNA, each strand of which is 2 nt longer than the other at the 3′-end. According to this structural feature, we aligned small RNAs to each other to find siRNAs meeting this criterion. A total of 184,498 tags were found to form RNA duplexes. These tags were potential siRNA candidates. The majority of them (58.52%) were 24 bp, indicating the function of DCL3 in this processing. Furthermore, we found 3933 siRNAs mapped on the expressed retrotransposons. Interestingly, 2296 siRNAs were mapped on *DX404336* and *DX403101*, and 1259 on *DX402782*. Their distribution on sequences was uneven (Figure 4). Using qRT-PCR, we examined the expression of the two retrotransposons regions—both were induced in 4-h sample and repressed in 5-d sample (Figure 5). 

### 3.5. Transcriptome-Wide Identification of miRNA Targets in Cotton

To gain insight into the functions of salt-regulated miRNAs, target genes were identified through a degradome sequencing approach. Three libraries, prepared from the CK, 4-h and 5-d samples were constructed. A total of 73,988,644 reads represented by 3,254,054 unique reads from the 5′-ends of uncapped and poly-adenylated RNAs were obtained (GEO: GSE69820). These sequences were compared with transcriptome sequencing data to identify the sliced targets for the known and novel miRNAs.

**Figure 6:**
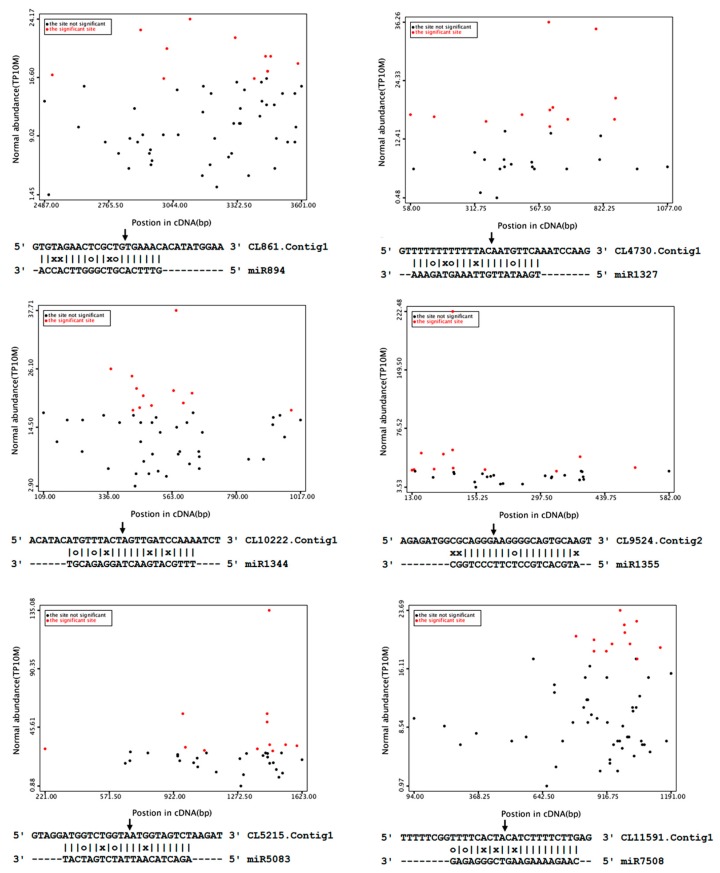
Target plot (t-plot) for target genes of less-conserved miRNAs. Arrows indicate the signatures corresponding to the miRNA cleavage site. Partial mRNA sequences of target genes aligned with the miRNAs show perfect matches (straight lines), G-U wobbles (circles).

**Figure 7:**
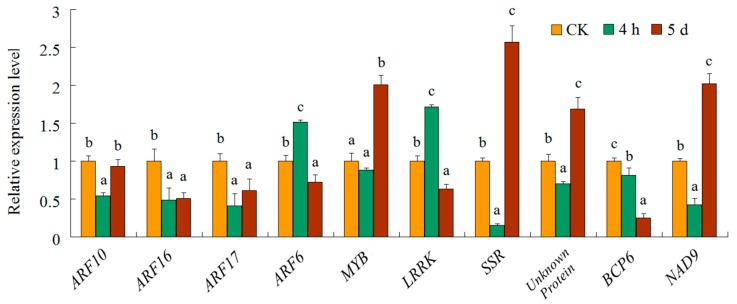
Using qRT-PCR, the expression profiles of ten target genes were examined under salt stress. There were three biological replicates and three technical replicates. The different letters of each target genes present on the columns indicate significant differences at *p* < 0.05 among the CK, 4-h and 5-d samples. Error bars represent standard error of the mean.

Based on degradome sequencing, a total of 31 target genes were identified for 20 cotton miRNA families (Table 3). The abundance of transcripts was plotted for each transcript. It is well-established that conserved miRNAs target conserved homologous genes in diverse plant species (Figure 6). In this study, 15 target genes for 10 highly or moderately conserved miRNAs were identified. These included five *auxin response factors* (*ARF*) targeted by miR160; two No Apical Meristem domain transcription factor (*NAC*) targeted by miR164; one *HD-Zip* protein targeted by miR165; one *ARF6* gene targeted by miR167; one *NF-YA3* gene targeted by miR169; one *AP2* targeted by miR172; one MYB targeted by miR319; one *growth-regulating factor* (*GRF*) gene targeted by miR396; and one *MYB* targeted by miR828. MYB proteins are characterized by their MYB domain. Interestingly, we found that two genes of MYB transcription factor family proteins emerged as the targets of miR319 and miR828. The divergence of miRNA target sites suggested that subgroups of the *MYB* gene family might be regulated by different miRNAs. To test the biological function of salt-regulated miRNAs, a negative-correlation expression test was undertaken for their target mRNAs using qRT-PCR. Among the 10 targets tested, eight showed a negative correlation of expression with their corresponding miRNAs (Figure 7). Negative correlations between miRNAs and target mRNAs did not occur in all cases. There was no significant difference between the expression levels of *ARF 16* (target of miR160) in the 4-h and 5-d samples. The expression of *NAD9*, a target of miR7505, was significantly down-regulated in 4-hr samples and was up-regulated in 5-d samples.

### 3.6. Cotton miR390/tasiRNA-ARF/ARF4 Pathway Analysis

Using a homology search approach, four tasiRNA-ARFs were identified in cotton (Table 4). Based on *Arabidopsis* tasiRNA-ARF nomenclature, they were named TAS3a D7(+), TAS3a D8(+), TAS3b D7(+), and TAS3b D8(+). They were identical to or had one nucleotide mismatch with their corresponding homologs in *Arabidopsis*. Transcriptome analysis was further used for the identification of *TAS3* loci. *TAS3a* and *TAS3b* were mapped on the *Unigene17307* and *Unigene12729* sequences, respectively. 

**Figure 8:**
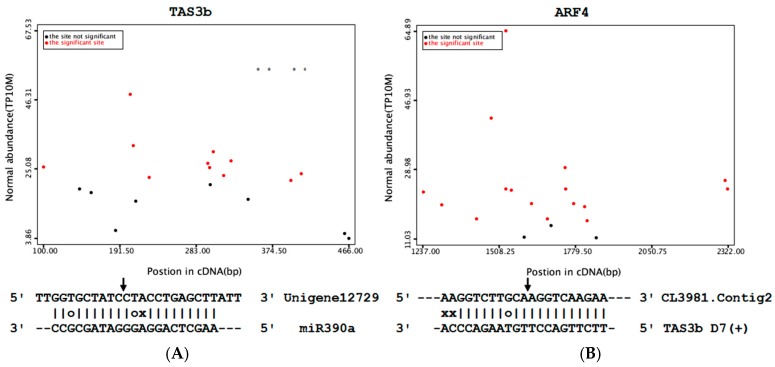
Target plot (t-plot) for cotton TAS3b (**A**) and ARF4 (**B**). Arrows indicate the signatures corresponding to the cleavage site. Partial mRNA sequences of target genes aligned with small RNA show perfect matches (straight lines), G-U wobbles (circles).

**Figure 9:**
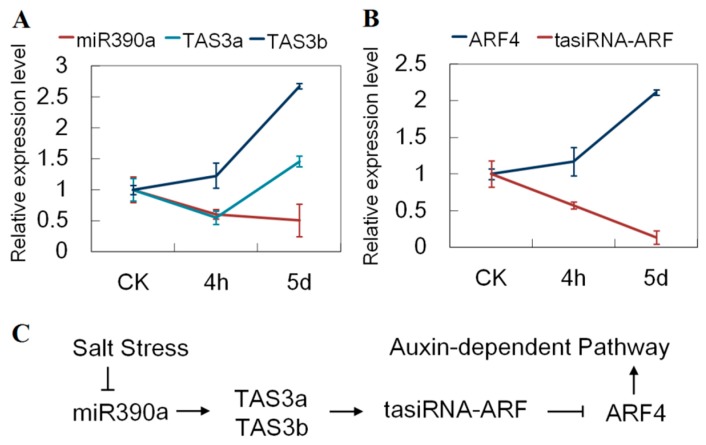
miR390/tasiRNA-ARF/ARF4 pathway analysis. Error bars represent standard error of the mean. (**A**) Relative expression levels of miR390 and TAS3 under salt stress; (**B**) Relative expression levels of tasiRNA-ARFs and ARF4 under salt stress; (**C**) A model for the role of the miR390/tasiRNA-ARF/ARF4 module under salt stress.

To investigate the molecular evolution of the two *TAS3* loci in *Gossypium* species, we also identified *TAS3* loci in *G. arboretum* and *G. raimondii*, the two diploids whose interspecific hybridization led to the tetraploid *G. hirsutum*. Each diploid *Gossypium* species had two *TAS3* loci, with the same sequences as in *G. hirsutum*, indicating the highly conserved nature of *TAS3* in *Gossypium*. TAS3 precursors had two recognition sites for miR390. The space between the two sites in Unigene17307 was 261 nt (12 phases); and was 174 nt (eight phases) in Unigene12729. However, our degradome sequencing results showed that only one target site was cleaved in both TAS3 precursor RNAs (Figure 8A). When the expression levels of miR390, TAS3a and TAS3b were monitored in cotton exposed to salt stress (Figure 9A), the miR390 levels reduced as the duration of exposure was extended. In contrast, the level of TAS3b increased. TAS3a level was firstly reduced, and then increased. Their negative correlation of expression supports the cleaving function of miR390 for TAS3, especially TAS3b. 

Transcriptome sequencing data was used to predict potential target genes of tasiRNA-ARFs, resulting in detection of six transcripts with one or two recognition sites. The potential target mRNAs showed near perfect complementarity to the 21-nt tasiRNA-ARFs, with <2 nt mismatches. Subsequently, the CDS database of *G. arboretum*, *G. raimondii* and *G. hirsutum* was used to examine the functional similarity of predicted target mRNAs. CotAD_43230, CotAD_76410 and CotAD_58764 were annotated as *ARF2* (Table 5). CotAD_00650, CotAD_64060 and CotAD_72124 were annotated as *ARF3*. CotAD_13694, CotAD_54309 and CotAD_12422 were annotated as *ARF4*. *ARF3* and *ARF4* contained two recognition sites. *ARF2* contained one single recognition site. All of the tasiRNA-ARF recognition sites were located in coding regions (Figure S4). Of these *ARF* genes, cleavage sites were identified in *ARF*4 in our degradome study. We examined the expression levels of *ARF4* and tasiRNA-ARF [TAS3b D7(+)] exposed to salt stress. As the duration of exposure increased, the tasiRNA-ARF level reduced and *ARF4* level increased (Figure 9B).

## 4. Discussion

In this study, we analyzed Na^+^ content and proline levels, and monitored the molecular responses of cotton plants exposed to sudden and gradual salt application. Na^+^-specific damage is associated with the accumulation of Na^+^ in the plant cell cytoplasm. The low increase rates of Na^+^ concentration at the beginning of salt shock suggested that ion stress was at a low level. The Na^+^ contents reached a high level at five days under long-term salt stress. It suggests that external salt concentration caused an ion imbalance under salt shock and Na^+^ concentrations accumulated with the increase of processing time under long-term salt stress conditions. The ionic stress component of salinity stress becomes gradually more severe after gradual salt application. Proline is known as a molecular chaperone to protect protein integrity and increase the enzyme activities under salt stress. It is produced from either glutamate or ornithine in plants, whereas in mature plants or during exposure to stress the glutamate pathway usually dominates [43]. In our study, proline levels in cotton leaves significantly increased under long-term salt stress. It seems that many genes involved in the accumulation of proline and other osmolytes should also be induced. Recent advances indicated that salt tolerance in plants is a qualitative and quantitative trait controlled by multiple genes [44,45]. It is essential to pay more attention to the dynamic activity of post-transcriptional and post-translational regulators in this field [7,46]. 

miRNA expression profiles in response to salt stress have been analyzed in a variety of plant species, including *Arabidopsis*, tobacco, rice, maize and poplar [7,16,24,47,48]. In this study, we monitored miRNAs and the expression of their target genes under salt shock and long-term salt stress. A total of 141 miRNAs exhibited altered expression. Among them, 20 and nine miRNAs showed consistent up- or down-regulation, respectively. Six miRNAs were specifically expressed in the 4-h sample. Three miRNAs were specifically expressed in the 5-d sample. It has long been apparent that environmental signals can modulate plant responses to detrimental conditions through changes in phytohormone concentrations [49]. The hormonal changes influence not only the normal biomass but also the adaptive response [50]. Patterns of gene expression were suggested to be different in response to salt shock and salt stress [2]. We studied the target genes of miRNAs by degradome sequencing. Most target genes of conserved miRNAs were transcription factors, playing crucial roles in gene regulation and involved in various aspects of plant growth and development. The majority of target genes of novel miRNAs were protein-coding genes involved in metabolism, protein modification and RNA or carbohydrate binding. miR160 and miR167 are well known as targeting members of the ARF family of transcription factors in the plant kingdom [51,52,53]. In our study, six miR160 genes and miR167g showed altered expression under salt stress. Using degradome sequencing, we detected fragments of cotton *ARF8*, *ARF10*, *ARF16* and *ARF17* mRNAs. They were precisely mapped to the end of each fragment of the nucleotide predicted for miR160- and miR167-directed cleavage. This analysis confirmed that the four cotton *ARFs* were post-transcriptionally regulated by miR160s and miR167s. ARF proteins can either activate or repress transcription, depending on the nature of the middle domain [54]. ARF10, ARF16 and ARF17 activate ARFs with Gln-Leu-Ser-rich middle regions, whereas, ARF6 represses ARFs with Pro-Ser-Thr-rich middle regions [55]. In our study, the expression of *ARF10*, *ARF16* and *ARF17* under salt stress was lower than under normal conditions. *ARF8* was firstly up-regulated and then down-regulated by salt stress. It seems that the altered expression of *ARF6*, -*10*, -*16* and -*17* by miR160 or miR167 improved the level of auxin, and in turn reset the developmental program and compensated for the biomass damage due to salt stress. 

tasiRNAs form a class of plant-specific endogenous small RNAs that function as miRNA-like post-transcriptional negative regulators [13]. In this study, two *TAS3* genes, *TAS3a* and *TAS3b*, with two near-identical 21-nt tasiRNA-ARFs that coaligned with the phases D7(+) and D8(+), were identified in three *Gossypium* species by homology search. The same sequences suggested high conservation of *TAS3* in *Gossypium* species. In *Arabidopsis*, it was recently demonstrated that miR390 cleaves the *TAS3* precursor for the production of tasiRNA-ARF, which could cleave the transcripts of *ARF3* (*ETT*) and its close homolog *ARF4* [56]. For cotton, cleavage sites for TAS3 precursors and *ARF4* were identified in our degradome sequencing results. In addition, cotton *ARF4* mRNAs showed a significant negative correlation with the corresponding miR390. These observations suggested that miR390, TAS3 tasiRNAs and their *ARF* targets defined a stepwise pathway in response to salt stress in cotton (Figure 9C). The degree of conservation observed in land plants suggests that the *tasiRNA-ARF* pathway plays a fundamental role in plant development. In dicots, theTAS3 tasiRNA-ARFs regulate leaf patterning, developmental timing and rate of lateral root growth [57]. Defects in this pathway could cause phenotypic abnormalities in flower gynoecium formation and leaf heteroblasty in *Arabidopsis* [58,59]. Adaxial localization of *tasiRNA-ARF* correlates with the restriction of *ARF3/4* localization in the abaxial domain during leaf development [58]. Among the 23 family members of ARFs in *Arabidopsis*, ARF3 and ARF4 are known to be transcriptional repressors [55]. As abaxial determinants, ARF3/4 have been suggested to be mediators for auxin signaling to partition adaxial and abaxial domains during leaf primordium formation [60]. Our results showed that cotton *ARF4* was dramatically up-regulated. Thus, it seems that auxin concentrations were repressed by the regulation of *tasiRNA-ARF*-dependent *ARF4* expression under salt stress. Among the available loss-of-function *Arabidopsis* mutants (for at least 18 *ARF* genes), only single mutants of *arf2*, *arf3*, *arf5*, *arf7*, *arf8* and *arf19* have phenotypes differing in growth or development [61,62,63,64]. Accordingly, further analysis of cotton *tasiRNA-ARFs* and their target genes could shed new light on their regulatory roles of auxin in salt stress response.

## 5. Conclusions

In this study, global transcriptional profiles of miRNA and other small non-coding RNAs were investigated in responses to salt shock and long-term salt stress. The salt-responsive miRNAs may function under salt stress in changing the normal level of regulated gene transcripts responsible for optimized developmental patterning, and thereby adjusting the durations of phenological phases. The identification of these small RNAs as well as elucidating their functional significance broadens our understanding of post-transcriptional gene regulation in salt stress response. 

## Figures and Tables

**Table 1:** Dataset summary of sequencing of small RNA, degradome and transcriptome libraries.

Category	Small RNA			Degradome			Transcriptome
	CK	4 h	5 d	CK	4 h	5 d	
Total reads	17,533,903	16,853,769	22,982,882	22,766,839	30,703,662	20,880,679	57,194,024
High quality	17,389,916	16,704,868	22,900,931	22,744,209	30,684,331	20,837,655	54,261,421
Clean reads	17,026,070	15,731,477	22,590,391	22,653,372	30,649,472	20,685,800	52,170,788
Unigene sequences	4,331,028	3,703,594	4,802,868	864,720	1,217,757	1,171,577	81,114

CK: Control Samples.

**Table 2:** List of the 24 novel miRNAs in cotton.

Name	Mature miRNA Sequence	Gene ID	LM (nt)	Arm	LP (nt)	G+C(%)	miRNA* Sequence	ΔG kcal/mol	MFEIs
miR1327	TGAATATTGTTAAAGTAGAAA	Unigene15286	21	3′	122	27.87	TCTACTTTAACAATATTCATA	−43.30	1.27
miR1335	TGAAGCTGCCACATGATCTAG	Unigene16386	21	5′	103	46.60	AGATCATGCTGGCAGCTTCAAC	−50.20	1.05
miR1336	TCTTTCCTACTCCTCCCATTCC	CL1064.Contig1	22	3′	105	39.05	AAGTGGGATGGGTGGAAAGATT	−44.50	1.09
miR1337	AATGGAGGAGTTGGAAAGATT	Unigene26231	21	5′	95	28.42	TTTCCAATTCCTCCCATTCCAC	−36.70	1.36
miR1338	AATGAGTTAGGCGAGAGGTTC	CL5909.Contig2	21	3′	169	31.36	CTCTGGTTTAACTCATTTGTA	−51.10	0.96
miR1339	AAAAGCAATGAAGAACGACCT	CL5831.Contig1	21	3′	102	38.24	GCTGCGTTCTTCATTGCTTAACA	−41.30	1.06
miR1340	ACCGGCCGGGGGACGGACTGGG	CL6985.Contig2	22	3′	119	65.55	CAGTCCCGAACCCGTCAGCTGC	−62.60	0.80
miR1341	CTTTTTATAGGATAGGGACTG	Unigene30026	21	3′	103	29.13	GTTCCTATCTTATTAAAAAGAA	−37.10	1.24
miR1342	TGAAGCTGCAAGATGATCTGA	CL8098.Contig4	21	5′	262	34.35	AGATCATGCTGGCAGCTTCAAC	−57.70	0.64
miR1343	CAGAGATCGTCAAAGCATATC	CL1118.Contig5	21	5′	94	37.23	TATGCTTTGACGATCTCTGAT	−60.90	1.74
miR1344	TTTGCATGAACTAGGAGACGT	Unigene10644	21	3′	105	54.29	CGGCTGCTAGTTCATGGATGCC	−45.60	0.80
miR1345	AAGCGGAAGCTGAATTAGTTG	Unigene14674	21	3′	76	43.42	GAAACTAATCGAGCTCCGTTTGA	−26.00	0.79
miR1346	GCTGCCATCTCATGCATTCGG	Unigene20900	21	5′	165	44.85	CGATGCATGGCATGGGAGCACCA	−59.10	0.80
miR1347	TACAGCTTTAGAAATCATCCCT	Unigene13628	22	5′	109	29.36	GGATGATTTCTAAAGCTCTAGA	−48.40	1.51
miR1348	AGTGTCTGGGTGGTGTAGTTGGT	Unigene4349	23	5′	83	50.60	CCATTCGAACACGGGCTCAGACATTT	−23.90	0.65
miR1349	TAACTTGTCTTCGCCCTTCTC	Unigene8023	21	3′	201	34.83	GAAGTGCATGGCAAGTTAGA	−48.70	0.70
miR1350	TGGCACGGCTCAATCAAATTA	Unigene7762	21	5′	89	38.20	ATTTGATTGAGCCATTCCAAC	−34.60	1.02
miR1351	ACTCATAATTTAGCAAAGTCG	Unigene8111	21	3′	129	28.68	TTGCTGGATTATGAGTCTAAT	−61.40	1.66
miR1352	AATGCTTGAGGTGATAGGTTCA	Unigene14827	22	5′	137	50.37	AACCATTGCCTCAAGCACTTG	−58.80	0.85
miR1353	AAGGCAAAGGAAGAAAAGAGTGA	CL9674.Contig1	23	5′	105	38.10	ACTCTTTTTTTCCTGCCTTGC	−45.90	1.15
miR1354	AAAGTGGATGAAATTTTTAGC	Unigene26528	21	3′	159	36.48	TAAAAATTTCATTCATTTCTA	−66.10	1.14
miR1355	ATGCACTGCCTCTTCCCTGGC	Unigene10226	21	3′	102	53.92	AACAGGCTGAGCATGGATGGA	−43.40	0.79
miR1356	AACTGTGAAGCTATAAGGTAT	Unigene19434	21	5′	110	37.27	ACTTTTGTTTCACATTAC	−23.90	0.65
miR1357	AAGCTGTTGATGGCCGGCATGA	CL9715.Contig1	22	5′	91	46.15	ATGCCTATCATCAGGAGACTCT	−30.30	0.72

LM: length of mature miRNAs; LP: length of precursor; MFEIs: minimal folding free energy indexes.

**Table 3:** Target genes of miRNAs identified in cotton.

miRNA Family	Target Gene	Alignment Score	Alignment Range	Cleavage Site	Category	Annotation	Abundance		
							CK	4 h	5 d
miR160	*Unigene5547 (CotAD_09605)*	4	378–398	389	2	ARF 10	361	254	207
	*CL11606.Contig2 (CotAD_08070)*	4	198–218	208	2	ARF16	361	54	207
	*CL11606.Contig1 (CotAD_76501)*	2.5	345–366	357	2	ARF16	2794	233	2249
	*CL1202.Contig4 (CotAD_55881)*	1	497–518	509	2	ARF17	420	83	394
	*CL1202.Contig2 (CotAD_36520)*	2	2–22	12	3	ARF17	476	83	307
miR164	*CL1985.Contig3 (CotAD_58864)*	4.5	19–39	30	2	NAC	0	61	0
	*CL1985.Contig5 (CotAD_43532)*	4.5	19–39	30	2	NAC	0	61	0
miR165	*CL795.Contig6 (CotAD_15207)*	4	93–113	103	3	Class III HD-Zip protein 4	111	0	63
miR167	*Unigene18486 (CotAD_40344)*	4.5	3–23	15	2	ARF6	45	0	125
miR169	*CL599.Contig2 (CotAD_06772)*	2	1095–1115	1105	3	Nuclear transcription factor Y subunit A-3	151	6	88
miR172	*CL799.Contig8 (CotAD_29570)*	0	761–782	773	2	AP2-EREBP	189	104	277
miR319	*CL11426.Contig1 (CotAD_73356)*	2	361–381	373	2	MYB	84	295	398
miR396	*Unigene30805 (CotAD_25614)*	2	2–22	12	2	GRF	0	5	45
miR828	*Unigene27368 (CotAD_00308)*	1	1–22	13	0	MYB	510	131	1060
miR894	*CL861.Contig1 (CotAD_07539)*	4	2507–2528	2521	4	Leucine Rich Repeat Kinase (LRRK)	0	3	35
miR1327	*CL4730.Contig1*	3.5	782–803	795	3	Simple Sequence Repeat Marker, mRNA sequence (SSR)	0	5	72
miR1344	*CL10222.Contig1*	4	429–450	438	4	Uncharacterized protein	47	2	46
miR1355	*CL9524.Contig2 (CotAD_06422)*	3.5	55–76	62	3	Copper binding protein 6 (BCP6)	58	0	14
miR8155	*CL11661.Contig1 (CotAD_51244)*	4	735–753	741	4	AUX/IAA	105	2	61
miR5083	*CL11334.Contig1 (CotAD_47266)*	3.5	334–356	341	2	Unknown protein	51	2	43
	*CL5215.Contig1 (CotAD_47976)*	3.5	1524–1546	1536	3	Nucleosome assembly protein	73	1	13
miR7484	*CL11292.Contig4 (CotAD_04736)*	4	2–24	17	2	Predicted protein	0	2	16
miR7502	*Unigene26285 (CotAD_22251)*	3.5	11–32	23	4	Trihelix	0	1	0
	*CL503.Contig1 (CotAD_21495)*	4.5	18–39	30	4	Allene Oxide Cyclase (AOC)	0	12	0
miR7505	*Unigene19150 (CotAD_63955)*	4	533–554	545	3	NADH-ubiquinone oxidoreductase subunit 9 (NAD9)	732	2	951
miR7508	*Unigene9225 (CotAD_26989)*	4.5	1–21	12	2	Pectin Methylesterase (PME2)	313	8	650
	*CL11591.Contig1 (CotAD_08283)*	4.5	1034–1054	1045	4	Translocon-associated protein subunit alpha	72	4	46
	*CL1882.Contig3 (CotAD_18999)*	4	16–36	28	4	Zinc finger family protein,	0	6	3
miR7512	*CL6.Contig3 (CotAD_04930)*	4.5	13–35	26	4	Uncharacterized protein	0	2	0
	*CL6.Contig4 (CotAD_04930)*	4.5	13–35	26	4	Uncharacterized protein	0	2	0
	*Unigene19759*	4	12–31	23	4	Alpha/beta-Hydrolases superfamily protein isoform	0	1	0

**Table 4:** Abundance of four tasiRNA-ARFs and genomic position of two *TAS3* genes in three *Gossypium* species.

Name	Sequences	Abundance			Genomic Position		
		CK	4 h	5 d	*G. raimondii*	*G. arboreum*	*G. hirsutum*
TAS3a D8(+)	TTCTTGACCTTGTAAGGCCTT	2	2	1	Chr9: 41,462,977–41,462,936	scaffold13100:41–82	At_chr3: 23,630,524–23,630,565
TAS3a D7(+)	TTCTTGACCTTGTAAGACCCC	228	122	91			Dt_chr8: 37,518,786–37,518,827
TAS3b D8(+)	TTCTTGACCTTGTAAGACCTT	816	897	541	scaffold493: 67,555–67,595	Ca8:60,478,108–60,478,068	Dt_chr11: 54,832,692–54,832,651
TAS3b D7(+)	TTCTTGACCTTGTAAGACCCA	11,718	11,060	7524			At_chr11: 5,189,802–5,189,761

**Table 5:** List of the *ARF2*, *ARF3* and *ARF4* in three *Gossypium* species and the target sites for tasiRNA-ARFs.

Name	*G. raimondii*			*G. arboreum*			*G. hirsutum*		
	Gene identifier	Target Sites		Gene Identifier	Target Sites		Gene Identifier	Target Sites	
ARF2	Cotton_D_10022860 Cotton_D_10033064	1328–1351 1331–1352		Cotton_A_03644 Cotton_A_01955	1325–1346 1331–1352		CotAD_43230 CotAD_76410 CotAD_58764	1307–1329 1331–1353 1331–1353	
ARF3	Cotton_D_10031398 Cotton_D_10033714	1254–1275 1269–1290	1464–1485 1479–1500	Cotton_A_11311 Cotton_A_03933	1272–1293 1320–1341	1482–1503 1530–1551	CotAD_00650 CotAD_64060 CotAD_72124	1254–1275 1281–1303 1104–1125	1464–1485 1491–1512 1314–1335
ARF4	Cotton_D_10015852 Cotton_D_10019010	1368–1389 1350–1371	1575–1596 1554–1575	Cotton_A_01738 Cotton_A_11048	1197–1218 1359–1380	1404–1425 1563–1584	CotAD_13694 CotAD_54309 CotAD_12422	1368–1389 1350–1371 1359–1380	1575–1596 1554–1575 1563–1584

## Supplementary Materials

The following are available online at www.mdpi.com/2073-4425/8/12/369/s1. Table S1: Primer pairs used in real-time quantitative RT-PCR, Figure S1: Distribution of small RNAs among different categories in three libraries, Figure S2: Mature and the predicted fold-back structures of newly identified miRNAs in cotton. Sequences of mature miRNAs are underlined, Figure S3: Dissociation Curve of seven miRNAs for Real-time quantitative PCR, Figure S4: Target sites in *ARF**2*, *ARF3* and *ARF4* genes of *G. arboreum* and *G. raimondii*. Target site was shown as straight lines.
