# Supplementary material for: Comparative Analysis of Cotton Small RNAs and Their Target Genes in Response to Salt Stress
Source: Genes (Basel). 2017 Dec 5;8(12):369. doi: 10.3390/genes8120369 (PMC5748687; doi:10.3390/genes8120369)

Ghr-miR1327

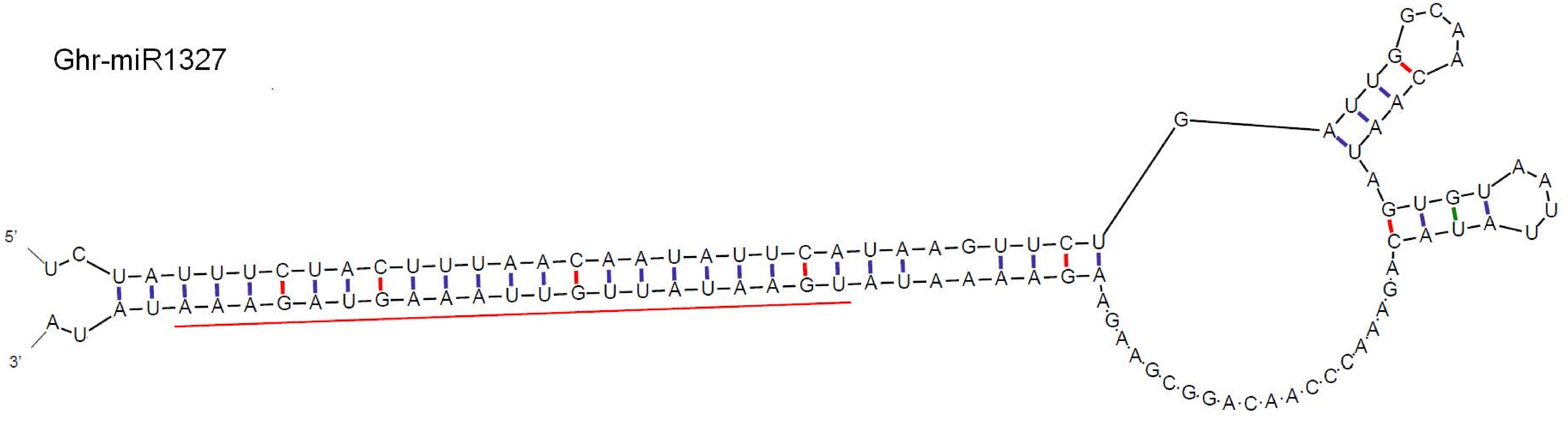

Ghr-miR1335

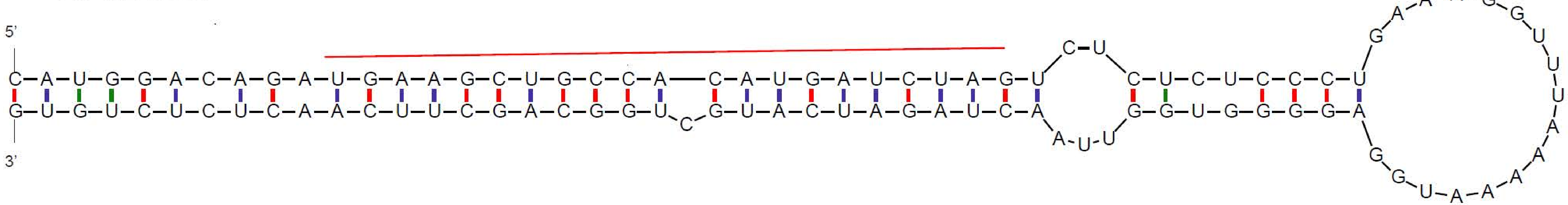

5'
C-A-U-G-G-A-C-A-G-A-U-G-A-A-G-C-U-G-C-C-A—C-A-U-G-A-U-C-U-A-G-U
C-U
C-U-C-U-C-C-C-U
G-A-A-A-G-G-U-U-U-U-A-A-A-A-U-G-G
G-U-G-U-C-U-C-U-C-A-A-A-C-U-U-C-G-A-C-G-G-U-C-G-U-A-C-U-A-G-A-U-C-A
A-U-U
G-G-U-G-G-G-A
3'

Ghr-miR1336

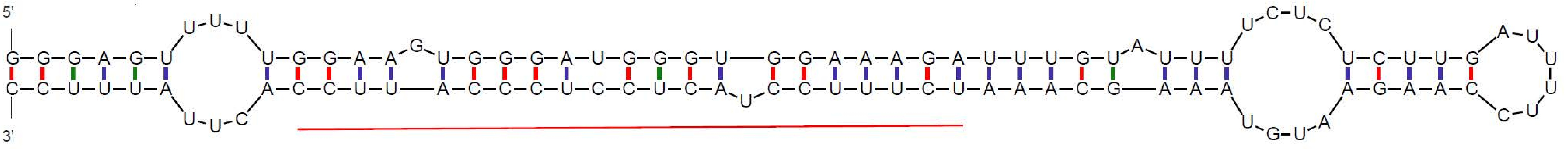

Ghr-miR1337

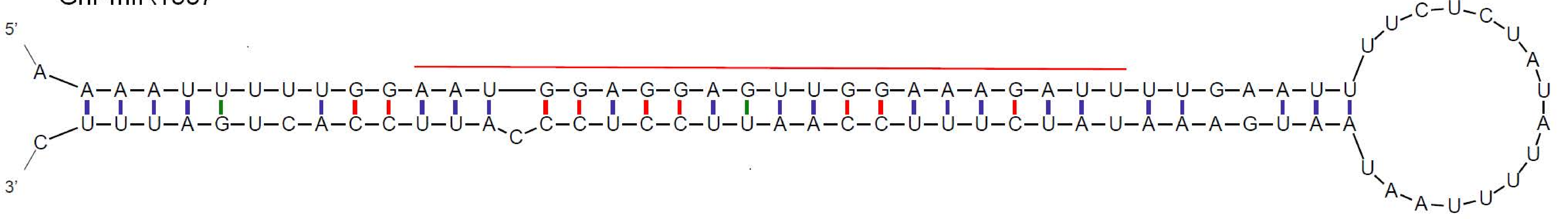
5'
3'
A
A-A-A-U-U-U-U-U-G-G-A-A-U—G-G-A-G-G-A-G-U-G-G-A-A-A-G-A-U-U-U-U-G-A-A-U-U
U-U-U-A-G-U-C-A-C-C-U-U-A-C-C-C-U-C-C-U-U-A-A-C-C-U-U-U-C-U-A-U-A-A-A-G-U-A-A
C
U-U-C-U-C-U-A-U-A-U-U-U-U-A-A-U

Ghr-miR1338

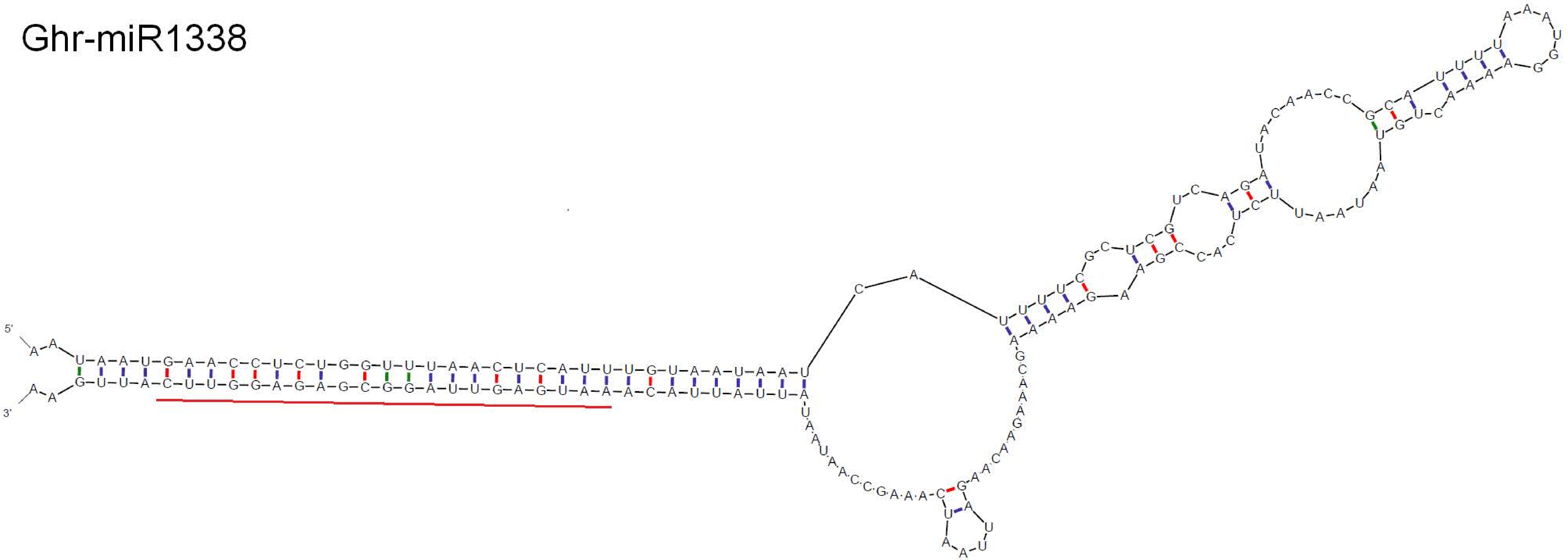

Ghr-miR1339

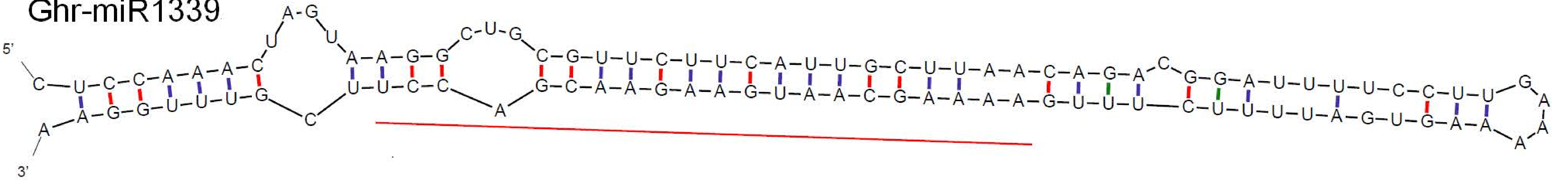

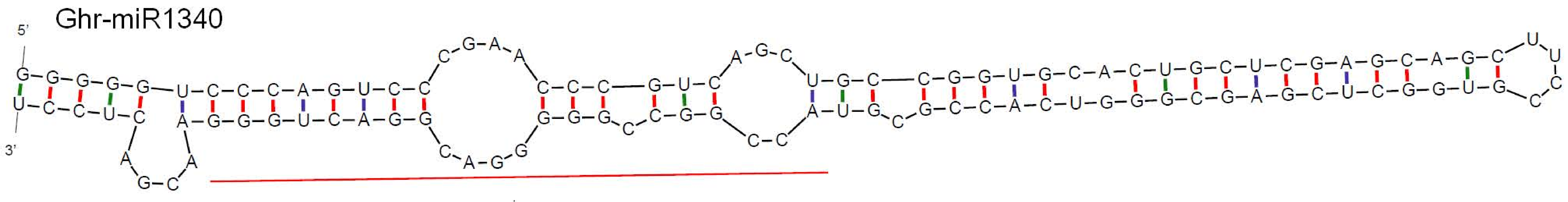

Ghr-miR1340
5'
3'

Ghr-miR1341

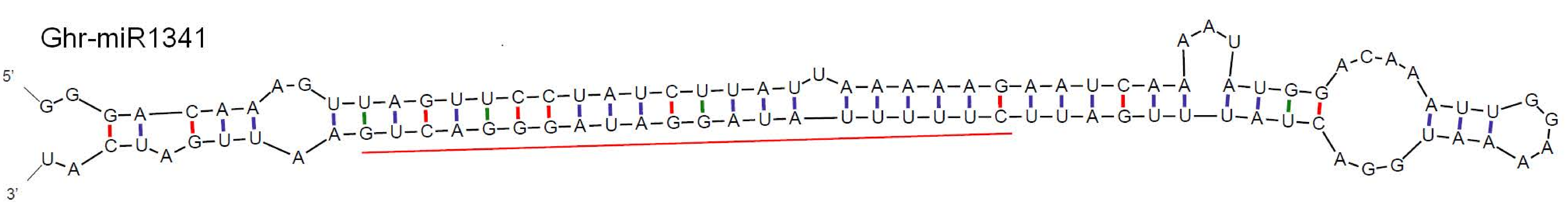

Ghr-miR1342

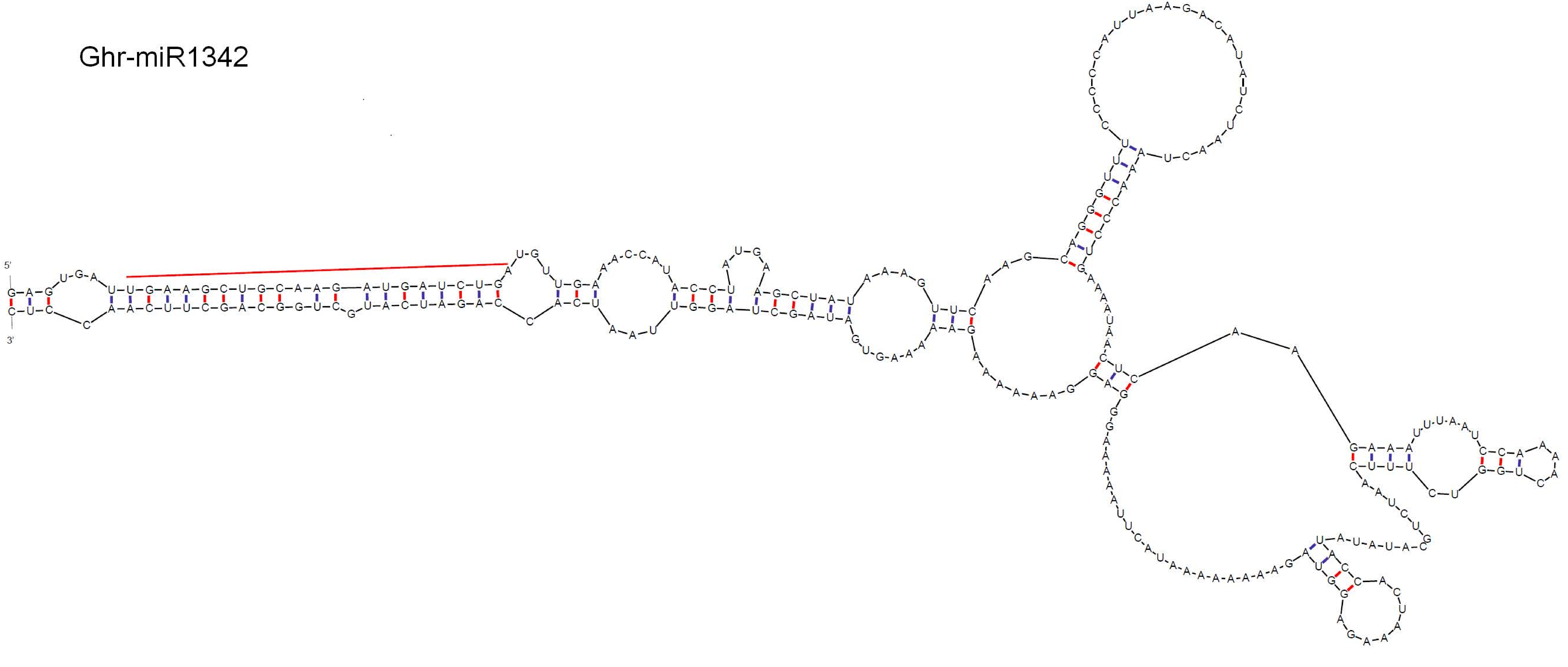

Ghr-miR1343

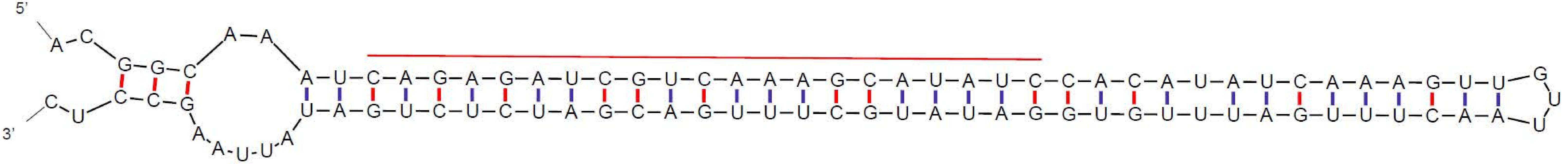

Ghr-miR1344

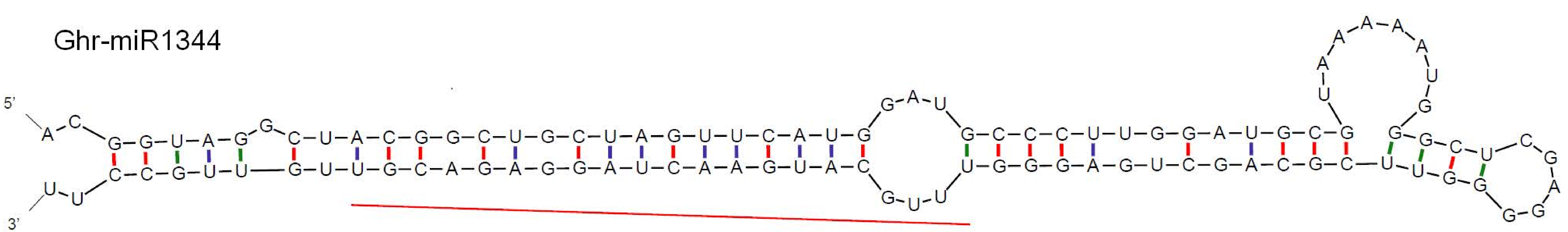

Ghr-miR1345

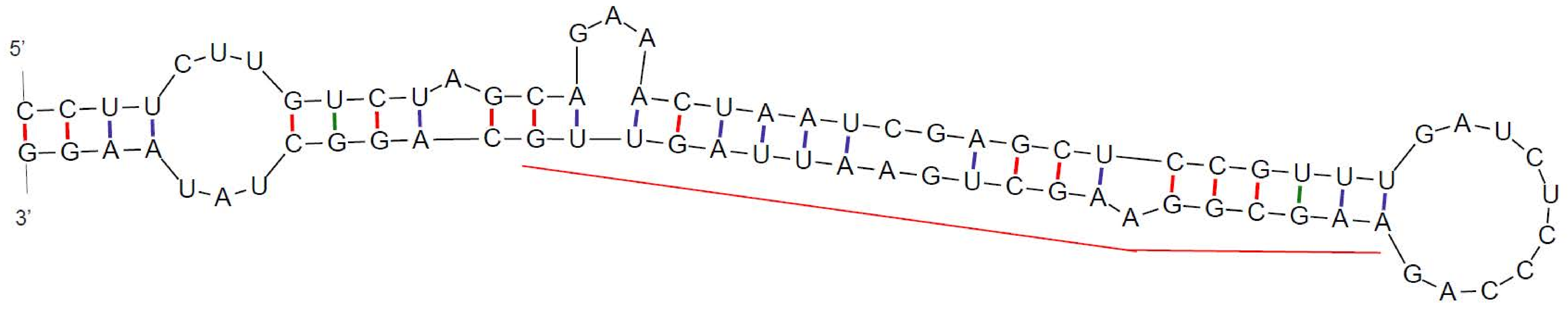

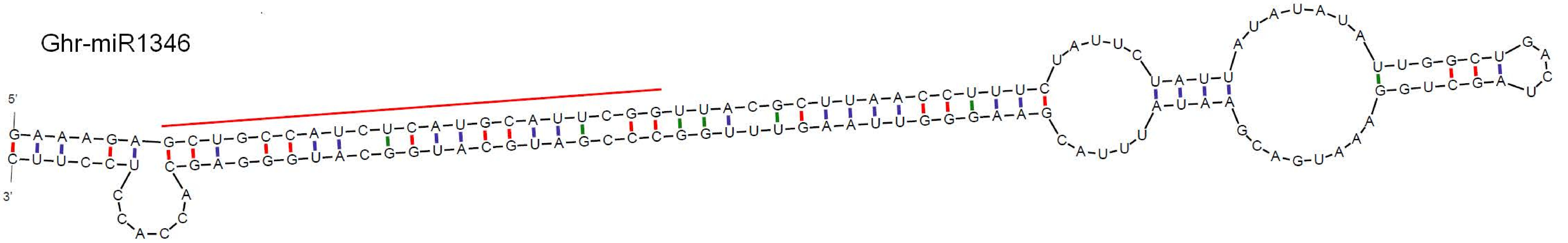
Ghr-miR1346
5'
3'

Ghr-miR1347

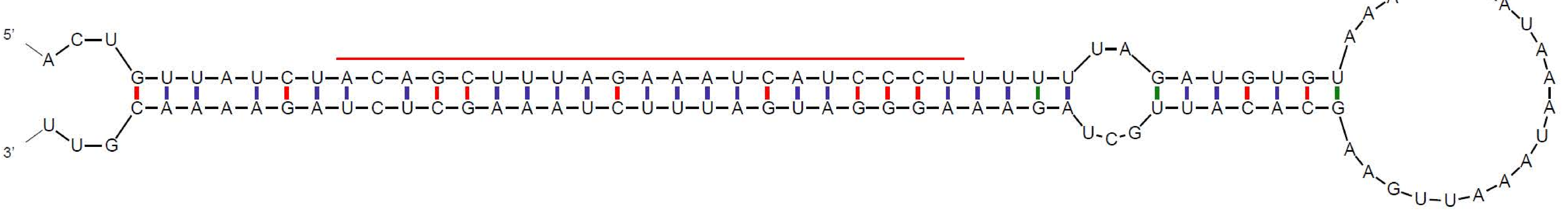
5’
3’
A-C-U
G-U-U-A-U-C-U-A-C-A-G-C-U-U-U-A-G-A-A-A-U-C-A-U-C-C-C-U-U-U-U-U
C-A-A-A-A-G-A-U-C-U-C-G-A-A-A-U-C-U-U-U-A-G-U-A-G-G-G-A-A-A-G-A
U-U-G
U-A
U-C-G
G-A-U-G-U-G-U
U-U-A-C-A-C-G
A-A-A-U-A-U-A-U-A-A-A-A-U
A-A-G-U-U-A-A-A

Ghr-miR1348

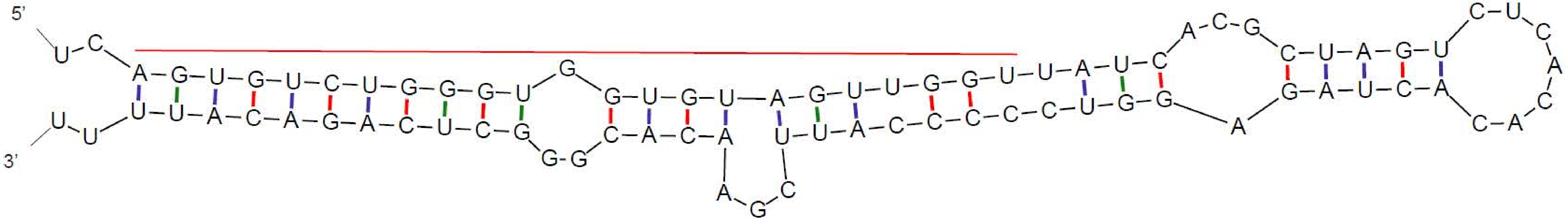

Ghr-miR1349

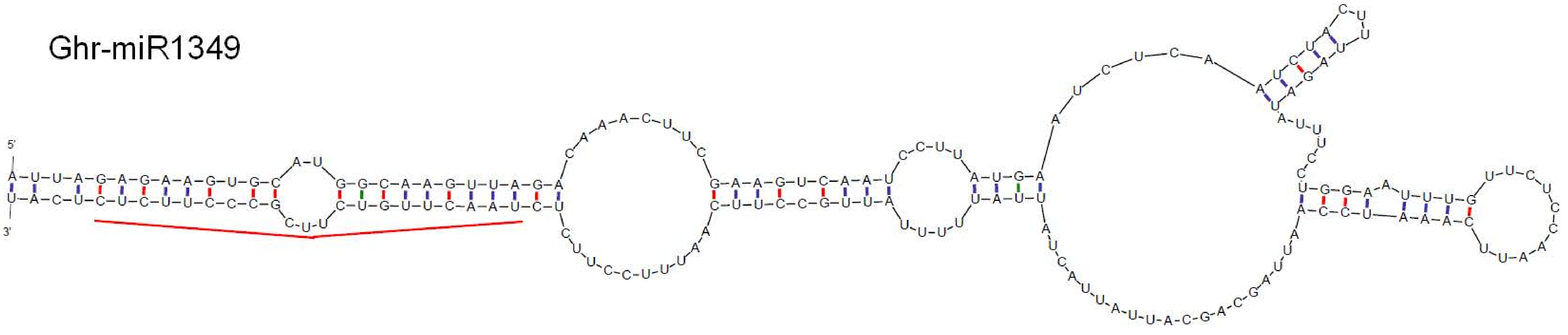

Ghr-miR1350

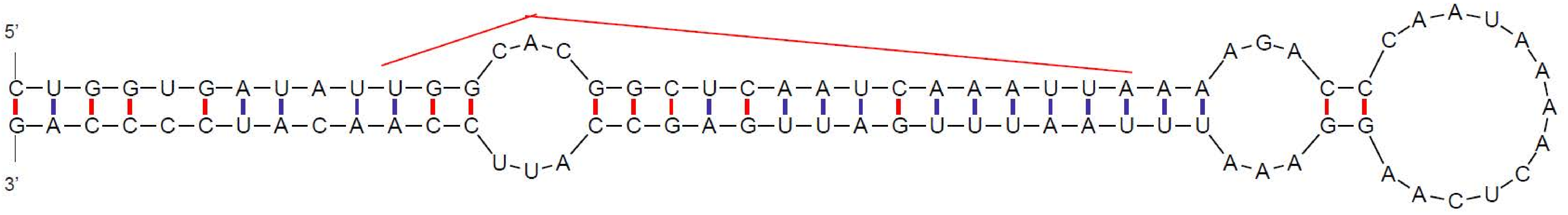

5'
C-U-G-G-U-G-A-U-A-U-U-G-G
C-A-C
G-G-C-U-C-A-A-U-C-A-A-A-U-U-A-A-A
A-G-A
C-C
C-A-A-U-A-A-A-A-A-C-U-C-A-A
G-G
A-A-A
G-A-C-C-C-C-U-A-C-A-A-C-C
U-U-A
C-C-G-A-G-U-U-A-G-U-U-U-A-A-U-U-U
3'

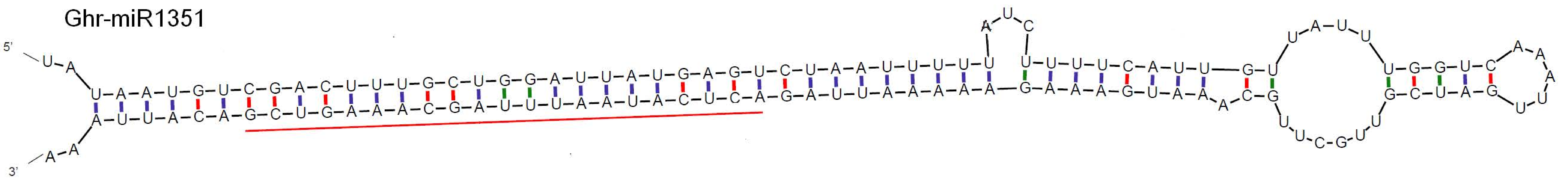
Ghr-miR1351
5'
3'

Ghr-miR1352

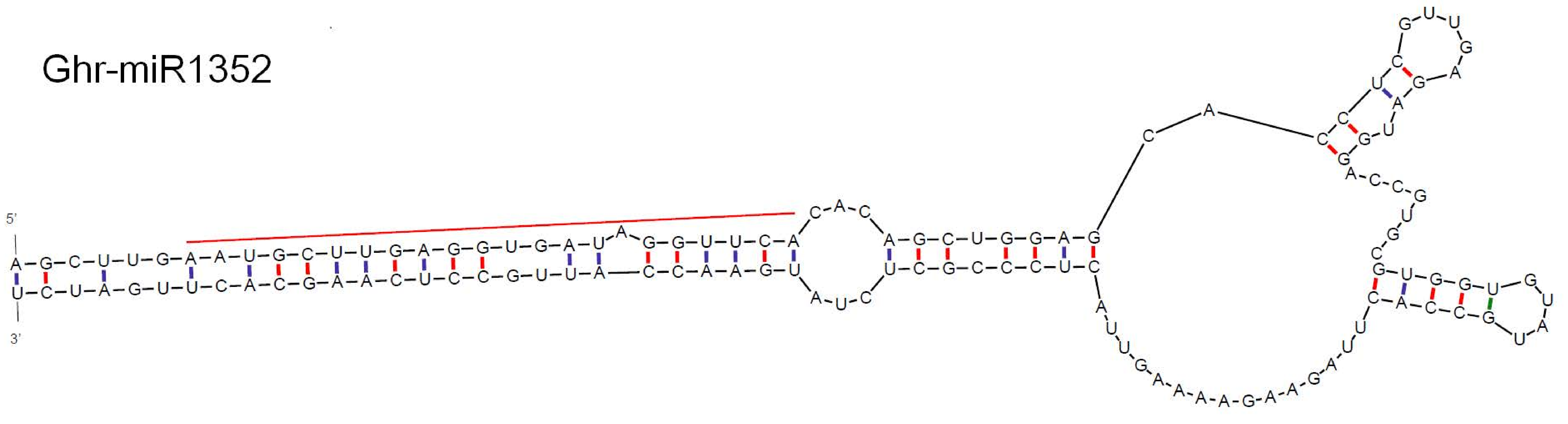

Ghr-miR1353

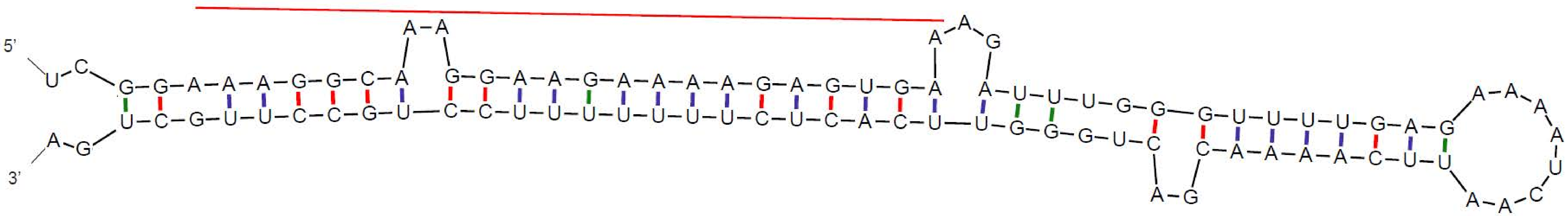

Ghr-miR1354

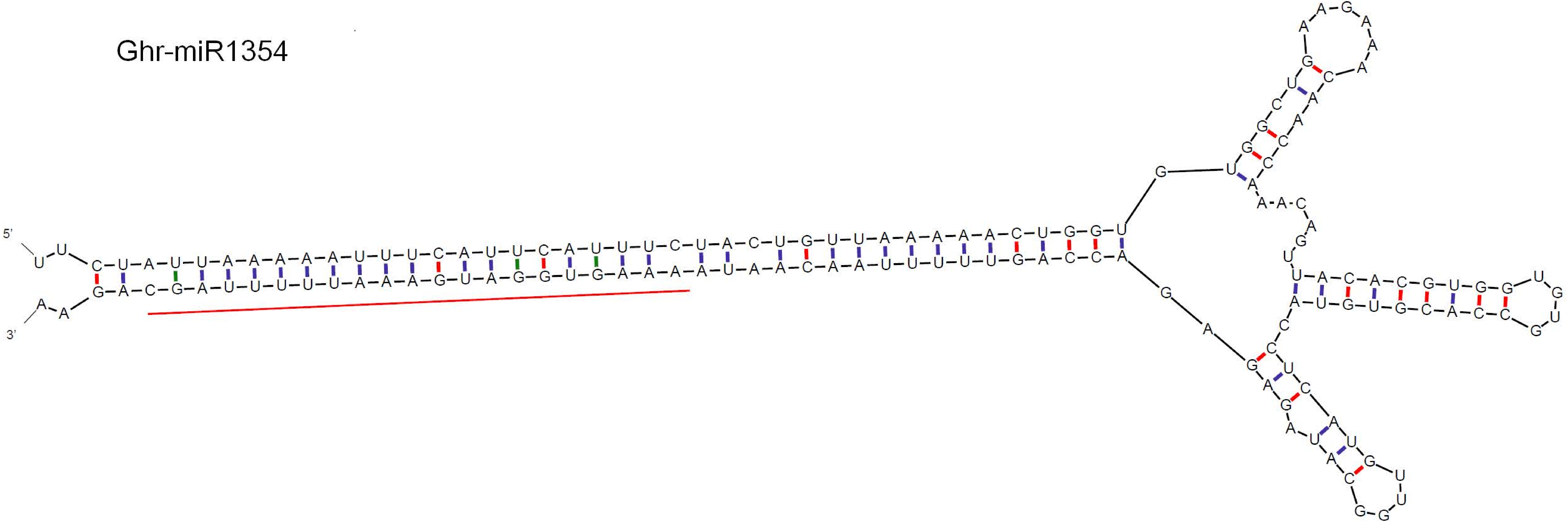

Ghr-miR1355

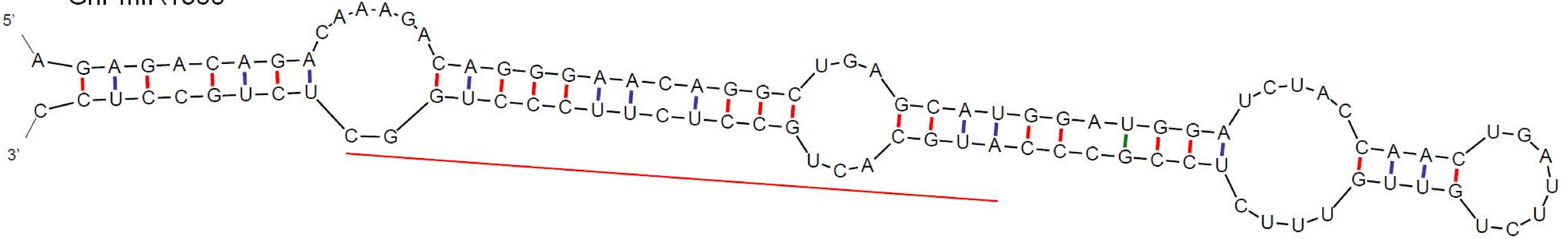

Ghr-miR1356

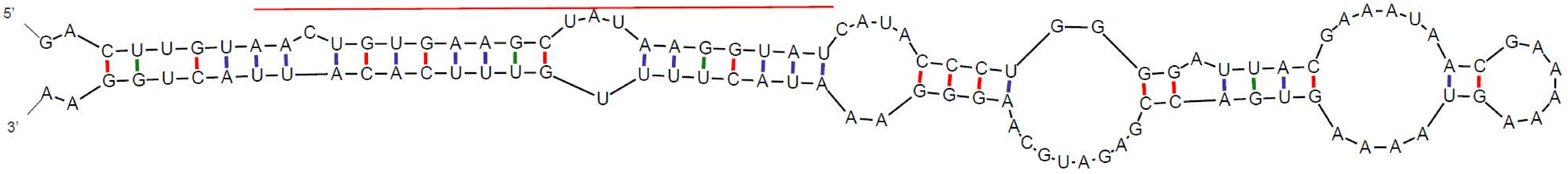

Ghr-miR1357

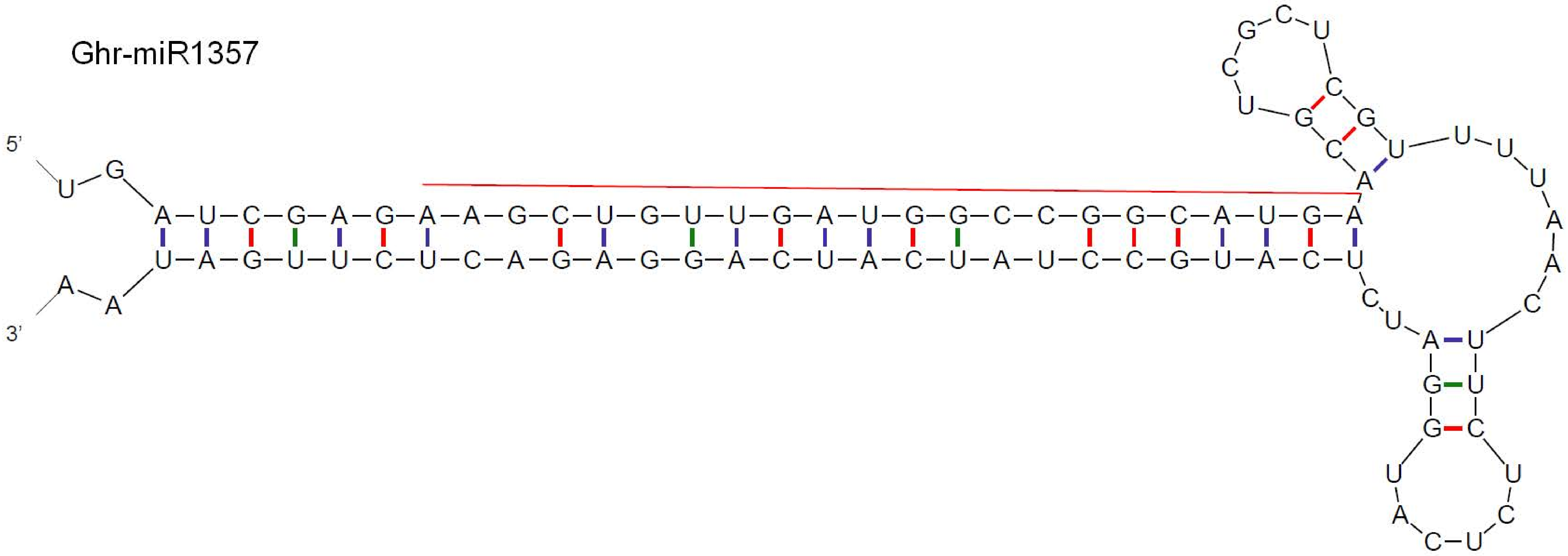

Supplement: Supplementary file 1 [file genes-08-00369-s001.zip › Figure S2 Mature and the predicted fold-back structures of newly identified miRNAs in cotton. .pdf]
