# Supplementary material for: Comparative Analysis of Cotton Small RNAs and Their Target Genes in Response to Salt Stress
Source: Genes (Basel). 2017 Dec 5;8(12):369. doi: 10.3390/genes8120369 (PMC5748687; doi:10.3390/genes8120369)
Supplement: Supplementary file 1 [file genes-08-00369-s001.zip › Figure S3 Dissociation Curve of seven miRNAs for Real-time quantitative PCR.pdf]

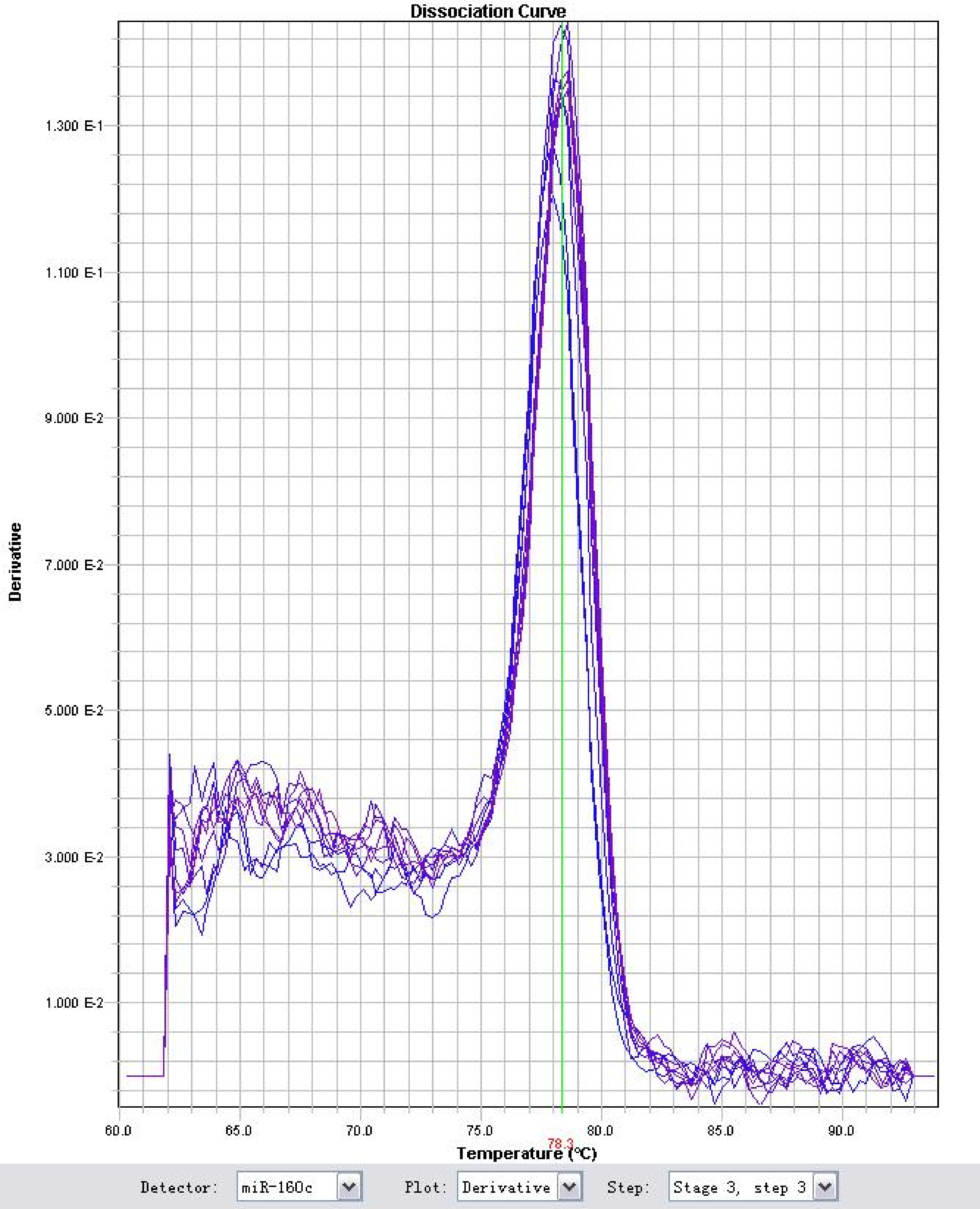
Dissociation Curve
1.300 E-1
1.100 E-1
9.000 E-2
7.000 E-2
5.000 E-2
3.000 E-2
1.000 E-2
Derivative
60.0
65.0
70.0
75.0
80.0
85.0
90.0
78.3
Temperature (°C)
Detector: miR-160c
Plot: Derivative
Step: Stage 3, step 3

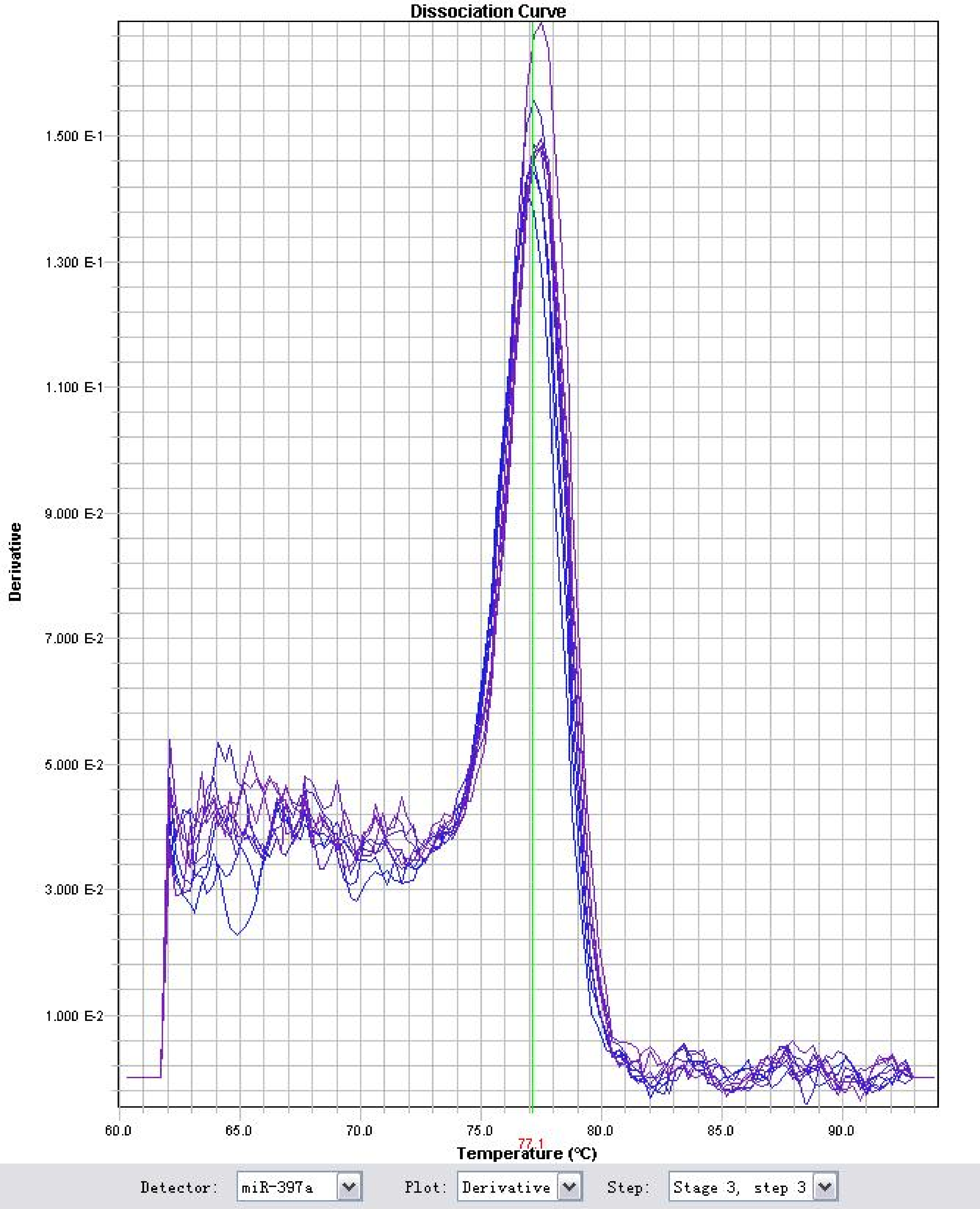

Dissociation Curve
Derivative
1.500 E-1
1.300 E-1
1.100 E-1
9.000 E-2
7.000 E-2
5.000 E-2
3.000 E-2
1.000 E-2
60.0
65.0
70.0
75.0
80.0
85.0
90.0
77.1
Temperature (°C)
Detector: miR-397a
Plot: Derivative
Step: Stage 3, step 3

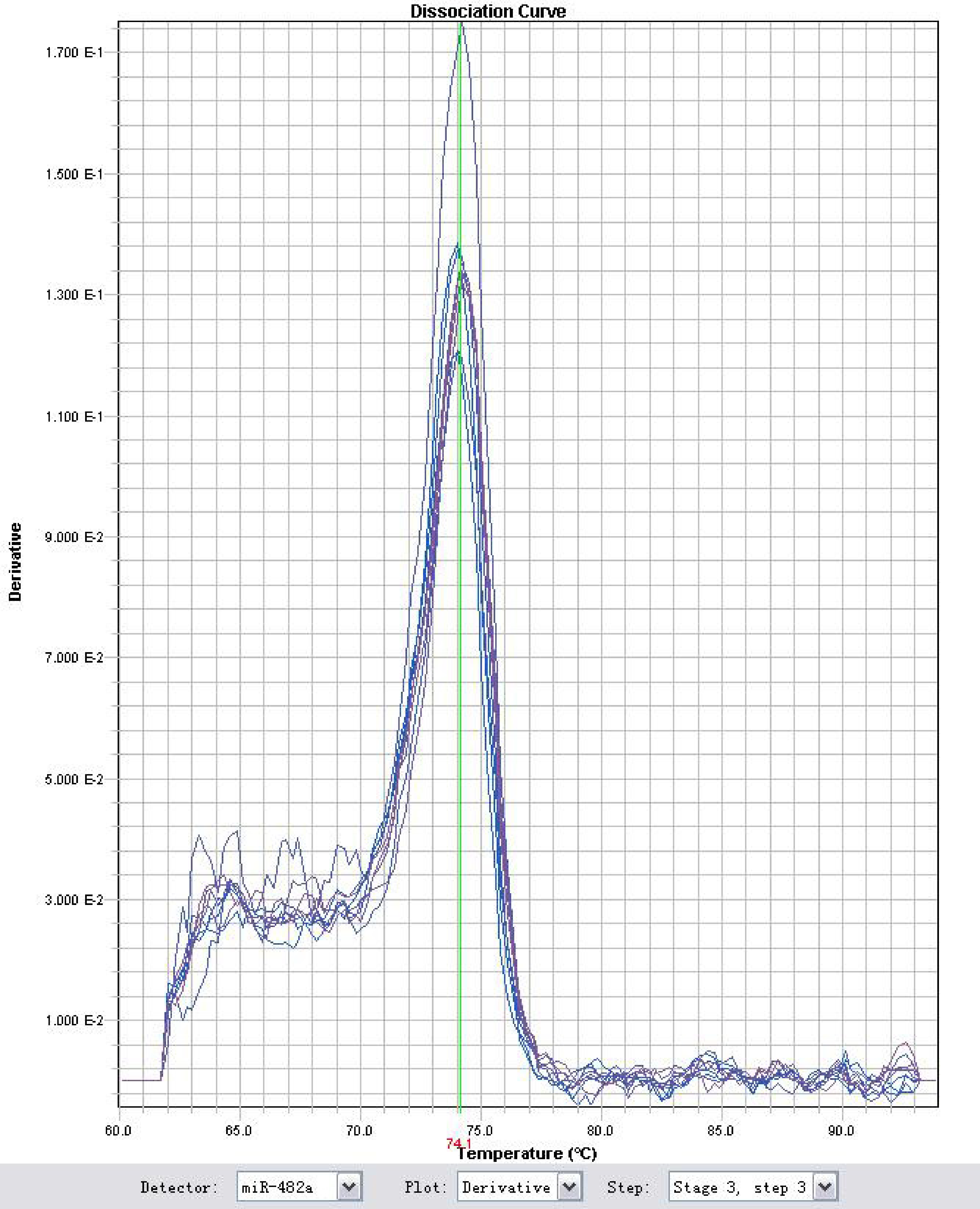

Dissociation Curve
1.700 E-1
1.500 E-1
1.300 E-1
1.100 E-1
9.000 E-2
7.000 E-2
5.000 E-2
3.000 E-2
1.000 E-2
Derivative
60.0
65.0
70.0
75.0
80.0
85.0
90.0
74.1
Temperature (°C)
Detector: miR-482a
Plot: Derivative
Step: Stage 3, step 3

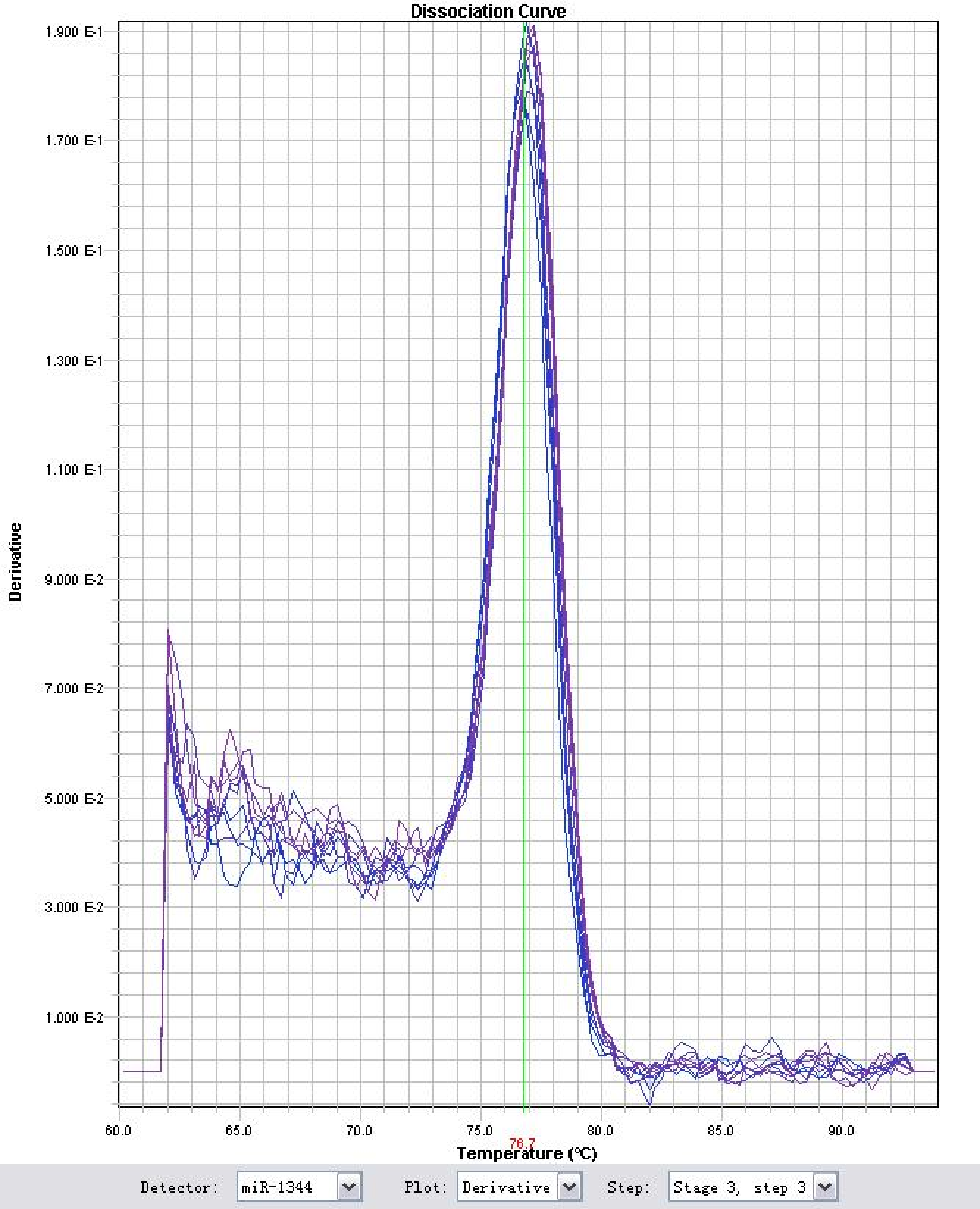
Dissociation Curve
1.900 E-1
1.700 E-1
1.500 E-1
1.300 E-1
1.100 E-1
9.000 E-2
7.000 E-2
5.000 E-2
3.000 E-2
1.000 E-2
Derivative
60.0
65.0
70.0
75.0
80.0
85.0
90.0
76.7
Temperature (°C)
Detector: miR-1344
Plot: Derivative
Step: Stage 3, step 3

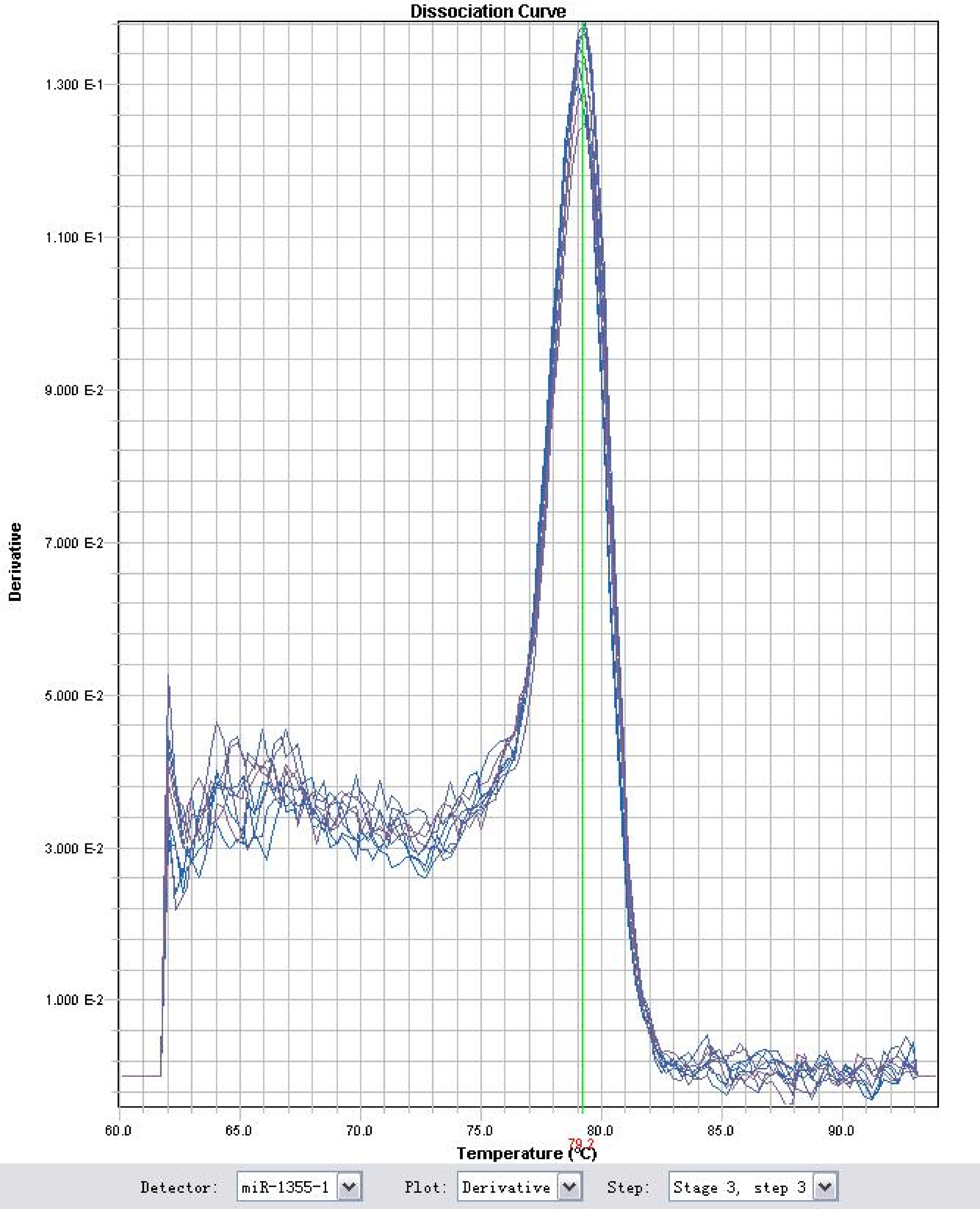

Dissociation Curve
Derivative
1.300 E-1
1.100 E-1
9.000 E-2
7.000 E-2
5.000 E-2
3.000 E-2
1.000 E-2
60.0
65.0
70.0
75.0
80.0
85.0
90.0
79.2
Temperature (°C)
Detector: miR-1355-1
Plot: Derivative
Step: Stage 3, step 3

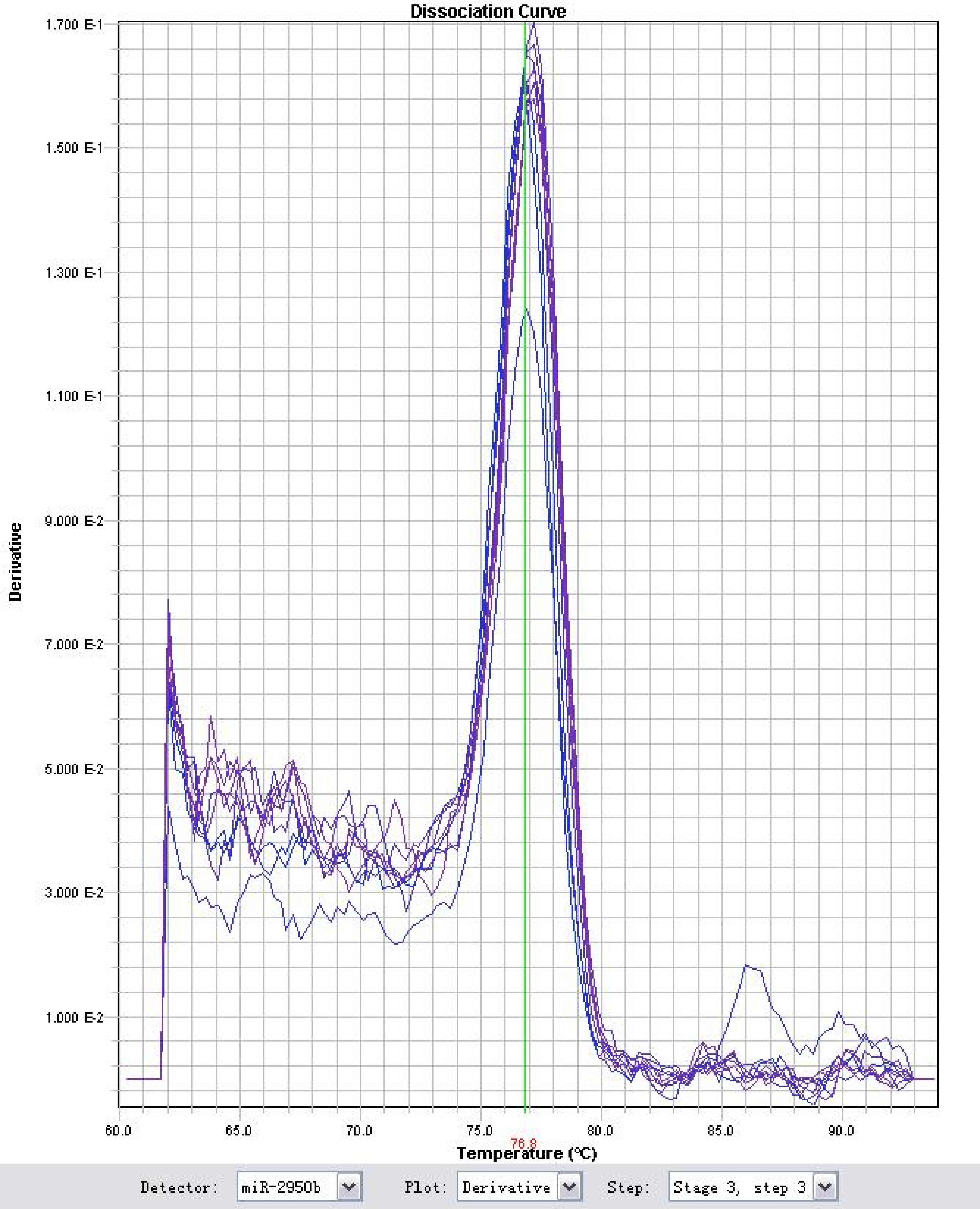

Dissociation Curve
1.700 E-1
1.500 E-1
1.300 E-1
1.100 E-1
9.000 E-2
7.000 E-2
5.000 E-2
3.000 E-2
1.000 E-2
Derivative
60.0
65.0
70.0
75.0
80.0
85.0
90.0
76.8
Temperature (°C)
Detector: miR-2950b
Plot: Derivative
Step: Stage 3, step 3

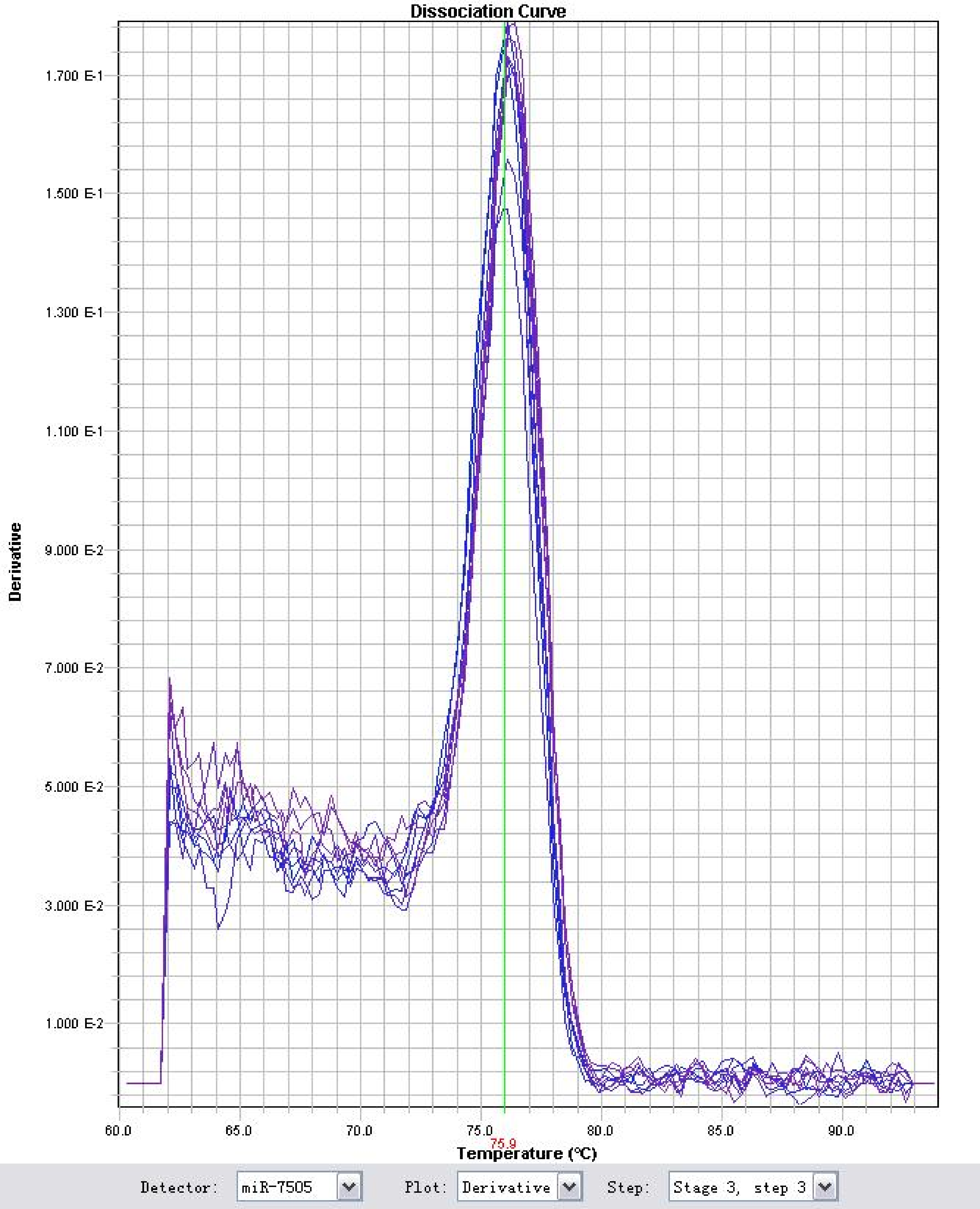

Dissociation Curve
Derivative
1.700 E-1
1.500 E-1
1.300 E-1
1.100 E-1
9.000 E-2
7.000 E-2
5.000 E-2
3.000 E-2
1.000 E-2
60.0
65.0
70.0
75.0
80.0
85.0
90.0
75.9
Temperature (°C)
Detector: miR-7505
Plot: Derivative
Step: Stage 3, step 3
