# Supplementary figures and images for: Comparative Analysis of Cotton Small RNAs and Their Target Genes in Response to Salt Stress
Source: Genes (Basel). 2017 Dec 5;8(12):369. doi: 10.3390/genes8120369 (PMC5748687; doi:10.3390/genes8120369)

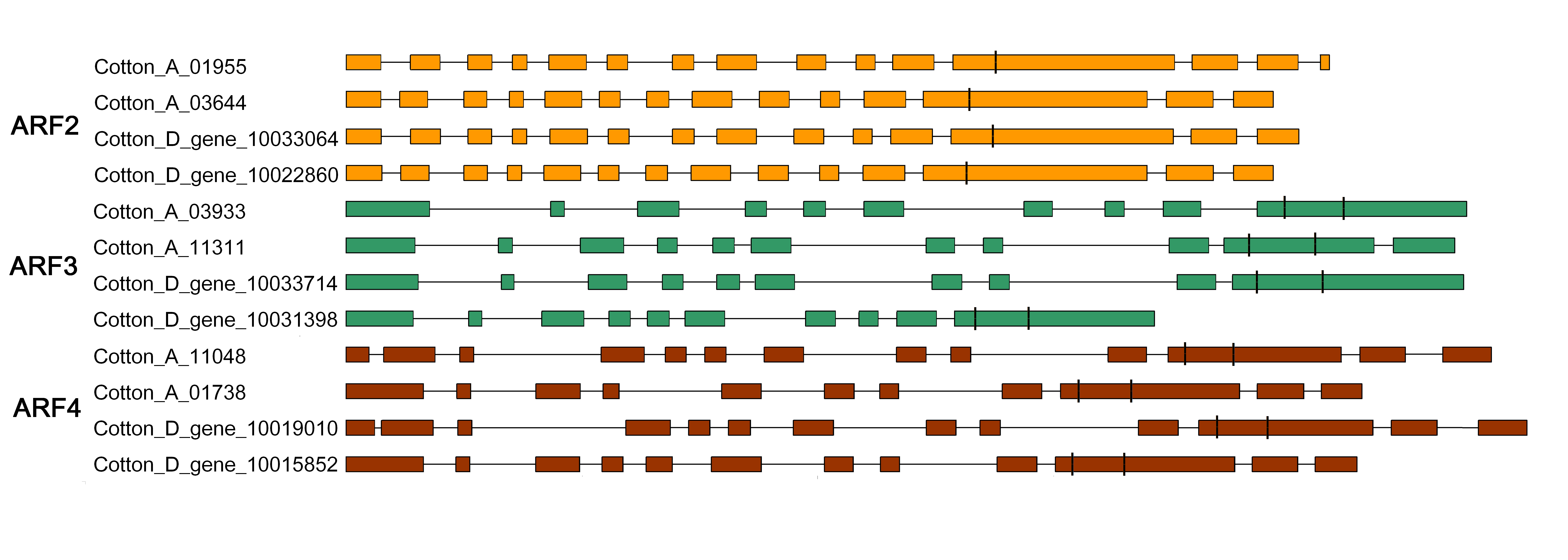

Supplement: Supplementary file 1 [file genes-08-00369-s001.zip › Figure S4 Target sites in ARF2, ARF3 and ARF4 genes of G. arboreum and G. raimondii..tif]

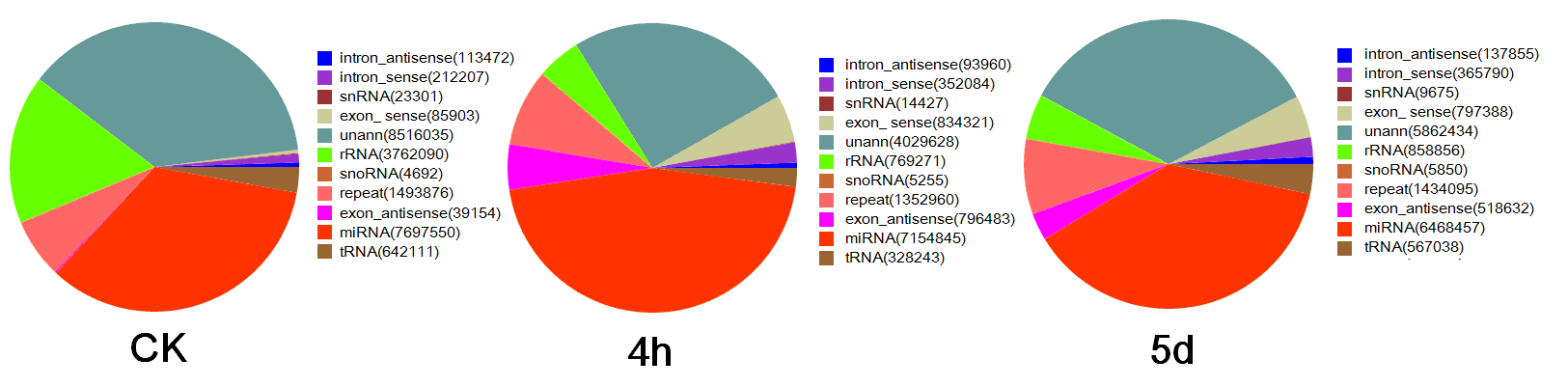

Supplement: Supplementary file 1 [file genes-08-00369-s001.zip › Figure S1 Distribution of small RNAs among different categories in three libraries.tif]
